# Development of prognostic models for Health-Related Quality of Life following traumatic brain injury

**DOI:** 10.1007/s11136-021-02932-z

**Published:** 2021-07-30

**Authors:** Isabel R. A. Retel Helmrich, David van Klaveren, Simone A. Dijkland, Hester F. Lingsma, Suzanne Polinder, Lindsay Wilson, Nicole von Steinbuechel, Joukje van der Naalt, Andrew I. R. Maas, Ewout W. Steyerberg, CENTER-TBI Collaborators

**Affiliations:** 1 Department of Public Health, Center for Medical Decision Making, Erasmus MC-University Medical Center Rotterdam, Rotterdam, The Netherlands; 2 Predictive Analytics and Comparative Effectiveness Center, Institute for Clinical Research and Health Policy Studies/Tufts Medical Center, Boston, USA; 3 Department of Medical Psychology and Medical Sociology, Georg-August-University, 37073 Göttingen, Germany; 4 University Medical Center Groningen, University of Groningen, 9713 GZ Groningen, The Netherlands; 5 Division of Psychology, University of Stirling, Stirling, FK9 4LA UK; 6 Department of Neurosurgery, University Hospital Antwerp and University of Antwerp, Edegem, 2650 Antwerp, Belgium; 7 Department of Biomedical Data Sciences, Leiden University Medical Center, PO Box 9600, 2300 RC Leiden, The Netherlands; 8 Department of Public Health, Erasmus MC University Medical Center, PO Box 2040, 3000 CA Rotterdam, The Netherlands

**Keywords:** Prognostic model research, Traumatic brain injury, Health-related quality of life, SF-36, QOLIBRI

## Abstract

**Background:**

Traumatic brain injury (TBI) is a leading cause of impairments affecting Health-Related Quality of Life (HRQoL). We aimed to identify predictors of and develop prognostic models for HRQoL following TBI.

**Methods:**

We used data from the Collaborative European NeuroTrauma Effectiveness Research in Traumatic Brain Injury (CENTER-TBI) Core study, including patients with a clinical diagnosis of TBI and an indication for computed tomography presenting within 24 h of injury. The primary outcome measures were the SF-36v2 physical (PCS) and mental (MCS) health component summary scores and the Quality of Life after Traumatic Brain Injury (QOLIBRI) total score 6 months post injury. We considered 16 patient and injury characteristics in linear regression analyses. Model performance was expressed as proportion of variance explained (*R*^2^) and corrected for optimism with bootstrap procedures.

**Results:**

2666 Adult patients completed the HRQoL questionnaires. Most were mild TBI patients (74%). The strongest predictors for PCS were Glasgow Coma Scale, major extracranial injury, and pre-injury health status, while MCS and QOLIBRI were mainly related to pre-injury mental health problems, level of education, and type of employment. *R*^2^ of the full models was 19% for PCS, 9% for MCS, and 13% for the QOLIBRI. In a subset of patients following predominantly mild TBI (*N *= 436), including 2 week HRQoL assessment improved model performance substantially (*R*^2^ PCS 15% to 37%, MCS 12% to 36%, and QOLIBRI 10% to 48%).

**Conclusion:**

Medical and injury-related characteristics are of greatest importance for the prediction of PCS, whereas patient-related characteristics are more important for the prediction of MCS and the QOLIBRI following TBI.

**Supplementary Information:**

The online version contains supplementary material available at 10.1007/s11136-021-02932-z.

## Background

Traumatic brain injury (TBI) is a leading cause of long-term impairments in functional, physical, mental, cognitive, and social domains [1]. These impairments are not restricted to severe cases, but are also known to occur frequently after moderate and mild TBI [2, 3]. Impairments can, for instance, be assessed using functional outcome scales [e.g., Glasgow Outcome Scale (Extended) (GOS(-E)] [4]. Although functional measurement scales are useful to portray functional problems, they do not capture the patient’s subjective experience of their sequelae and wellbeing in daily life [5].

Therefore, there has been growing interest in Health-Related Quality of Life (HRQoL) in TBI research. HRQoL focuses on an individuals’ perception of how a disease and its treatments affect the physical, mental, and social aspects of their life [6]. Previous studies confirmed that long-term impairments following TBI affect (HR)QoL [7–16]. To assess HRQoL two types of instruments are available: generic and condition-specific instruments [6]. Generic instruments, such as the Short Form-36 (SF-36) [17], do not take into account diseases or particular conditions and allow comparison with healthy individuals, as well as various health states or conditions. It has been argued that generic HRQoL instruments may not be sensitive enough to detect key issues in TBI, such as cognitive dysfunctions and psychological issues [6, 18]. A TBI-specific instrument, such as the Quality of Life after Traumatic Brain Injury (QOLIBRI) [19, 20], may, therefore, be complementary.

Outcomes following TBI depend on patient and injury characteristics, mechanisms of trauma, patient response, the social environment, and the quality of care provided [21–23]. Prognostic models predict the outcome of a patient based on characteristics at presentation and are important to help clinicians provide reliable information to patients and relatives [24]. It would be particularly helpful if poor HRQoL outcomes could be anticipated as these predictions could support clinicians in identifying patients who might benefit from close follow-up and early interventions. Although high-quality and well-validated models exist to predict functional outcome following moderate and severe TBI [25], prognostic models for HRQoL following TBI have not been developed yet. Furthermore, efforts have been made to identify predictors of HRQoL following TBI [11, 12, 14, 26–31], but they are dispersed throughout the literature. Therefore, we aimed to identify predictors and develop prognostic models for HRQoL following mild, moderate, and severe TBI.

## Methods

### Study population

We analyzed patients included in the Collaborative European NeuroTrauma Effectiveness Research in Traumatic Brain Injury (CENTER-TBI, version Core 2.1) study. This is a prospective, multicenter, longitudinal, observational study [32, 33]. Data were collected for patients with a clinical diagnosis of TBI and an indication for computed tomography (CT), presenting within 24 h of injury in one of the 58 participating centers. Participants were recruited from December 2014 to December 2017 in 18 countries across Europe and Israel.

For model development, patients were included if they were aged ≥ 18 years and completed the SF-36v2 or QOLIBRI at 6 months post injury.

Data for the CENTER-TBI study were entered on the Quesgen e-CRF (Quesgen Systems, Inc., USA), hosted on the International Neuroinformatics Coordinating Facility (INCF) platform and extracted via the INCF Neurobot tool (INCF, Sweden). Informed consent was obtained from all participants according to local and national requirements.

### Candidate predictors

Candidate predictors of HRQoL following TBI were selected based on literature, and included initial severity (Glasgow Come Scale) [12, 26, 27], age [28], sex [11, 28, 29], socioeconomic status [30], social support [28–31], pre-injury substance abuse [26, 28], and pre-injury mental health problems (e.g., anxiety, depression) [29, 34]. Additionally, major extracranial injury (MEI), injury cause, pre-injury health status, the presence of intracranial traumatic abnormalities, ongoing mental health problems, and 2 week HRQoL assessment were indicated by experts as potential predictors of HRQoL following TBI.

Ongoing mental health problems were assessed through scores for depression (PHQ9), anxiety (GAD7), and post-traumatic stress disorder (PCL5) at 2 weeks post injury. Socioeconomic status was assessed through type of education and type of employment. Social support was assessed through living arrangement. TBI severity was categorized into mild, moderate and severe based on the Glasgow Coma Scale (GCS) at admission. TBI was considered mild in patients with GCS 13–15, moderate in patients with GCS 9–12, and severe in patients with GCS of 3–8 [35]. MEI was defined as an Abbreviated Injury Scale (AIS) ≥ 3 on any extracranial domain of the scale [36]. Pre-injury health status was assessed with the American Society of Anesthesiologists- physical status classification system (ASA-PS); patients are categorized as ‘normal healthy patient’, ‘mild systemic disease’, ‘severe systemic disease’, or ‘severe systemic disease that is a constant threat to life’. The categories ‘severe systemic disease’ and ‘severe systemic disease that is constant threat to life’ were combined. The presence of intracranial traumatic abnormalities was assessed through the first computed tomography (CT) scan after injury, and indicates whether any of the 12 following abnormalities was present: mass lesion, hematoma, epidural hematoma, acute or subacute subdural hematoma, subdural collection mixed density, contusion, TAI, traumatic subarachnoid hemorrhage, intraventricular hemorrhage, midline shift or cisternal compression. The candidate predictors were assessed at admission within 24 h, except for early HRQoL assessment and ongoing mental health problems, which were conducted 2 weeks post injury.

Missing predictor values were imputed with 100 iterations with multiple imputation using the *mice* package [37]. All candidate predictors, injury severity score, and HRQoL outcomes between 2 weeks and 12 months were included in the imputation model.

### Outcome assessments

The primary outcomes were the physical (PCS) and mental (MCS) component summary scores from the Short Form-36v2 (SF-36v2) and the Quality of Life after Traumatic Brain Injury (QOLIBRI) total score at 6 months post injury. The SF-36v2 is a 36-item patient-reported outcome, which assesses multiple components of HRQoL: PCS; physical functioning, role limitations due to physical health, bodily pain, general health perceptions, vitality, MCS; social functioning, role limitations due to emotional health, and general mental health. Norm-based *T*-scores (standardized to mean 50 and SD of 10) were calculated for the PCS and MCS [17].

The QOLIBRI is a 37-item patient-reported outcome, consisting of four subscales assessing satisfaction with aspects of life (cognition, self, daily life and autonomy, and social relationships) and two subscales that concern how bothered the person is by difficulties (emotions, and physical problems) [19, 38].

### Data analyses

Descriptive statistics were presented as medians [interquartile range (IQR)] or frequencies (percentage) for the predictors and HRQoL data. Differences in patient- and injury-related characteristics between responders, those who completed the SF-36v2 or QOLIBRI between 2 weeks and 12 months post injury, and non-responders were compared using independent sample *t* tests (continuous) or *χ*^2^ tests (categorical).

We used linear regression analyses to quantify the relationship between predictors and the SF-36v2 PCS and MCS and the QOLIBRI total score at 6 months post injury. Model performance was expressed as proportion of variance explained (*R*^2^). For the continuous predictors—age and GCS—we assessed non-linearity with spline functions.

For each outcome, three prognostic models were defined: (I) the full model included all candidate predictors; (II) the extended model included a reduced set of predictors based on the Akaike information criteria (AIC); and (III) the core model included the three predictors with the largest partial *R*^2^. We also explored the incremental value of HRQoL assessment and mental health problems at 2 weeks post injury for the prediction of the PCS, MCS and QOLIBRI total score. Incremental value was assessed by the difference in *R*^2^ between the model with the additional predictors and the model without the additional predictors. Additionally, we explored the relationship between GCS (3–15) and all other predictors with interaction terms in multivariable analyses. Associations between predictors and outcome measures were presented with estimates of the regression coefficients and their 95% confidence interval (CI).

We assessed model performance through proportion explained variance (*R*^2^), and a bootstrapping procedure to reduce optimistic model performance estimates. Forty bootstrap samples were taken from the original dataset by sampling *X* entries equal to the sample size of the original cohort with replacement. Performance of the model that was fitted on the bootstrap sample was evaluated both in the bootstrap sample and the original cohort and the difference indicated the optimism in performance [24].

Five sensitivity analyses were performed. First, the models were fitted for the PCS, MCS, and QOLIBRI total score for a subset of patients who completed the questionnaires individually or together with a relative, friend or caregiver, therefore, proxy responses were excluded. Second, the models were fitted for the PCS, MCS and QOLIBRI total score at 3 months rather than 6 months post injury. Third, instead of only selecting patients with available 6 months outcome, the models were also fitted with additional imputed 6 months outcome whenever 3 or 12 months outcomes were available. Fourth, analyses were performed in subgroups of TBI severity—mild versus moderate and severe. Fifth, the models were fitted for impaired SF-36 PCS and MCS (< 40) and QOLIBRI total scores (< 60) [39].

Analyses were performed with R statistical software 3.6.0 [40]. We used the *rms* package to fit the regression models [41]. Modeling results were reported in accordance with the TRIPOD guidelines [42].

## Results

### Study population

We included 2666 adult patients who completed the SF-36v2 or the QOLIBRI between 2 weeks and 12 months post injury (Supplementary Fig. 1). Patients had a median age of 51 years (IQR = 33–65) (Table 1). More than half (65%) of patients were male, and most (74%) were diagnosed with mild TBI (GCS 13–15). A third (34%) had major extracranial injury. More than half (53%) were employed, and 24% were retired. About 10% had pre-injury mental health problems. Moreover, less than half of the patients (42%) experienced pre-injury comorbid health issues.

**Table 1:** Patients’ demographic and injury characteristics

Characteristics	Responders^a^ (*n* = 2666)	Non-responders^b^ (*n* = 1097)	*p*-value*
Demographics			
Age (18–95) (median, [IQR])	51 [33–65]	47 [30–65]	> .05
% Male sex	65 (1729)	71 (773)	< .05
Living arrangement (*N*, %)			
Together	2093 (79)	834 (76)	< .05
Missing (%)	3 (0.1)	8 (1)	
Highest level of education			< .001
None or primary school	321 (12)	124 (11)	
Currently in or with diploma/degree oriented program	555 (21)	199 (18)	
Secondary school/High school	820 (31)	305 (28)	
College/University	666 (25)	141 (13)	
Missing (%)	304 (11)	328 (30)	
Employment status			< .001
Yes	1410 (53)	453 (41)	
No	447 (17)	210 (19)	
Retired	643 (24)	243 (22)	
Missing (%)	166 (6)	191 (17)	
Employment type (*N*, %)			< .001
Working	1410 (53)	453 (41)	
Looking for work, unemployed	145 (5)	74 (7)	
Unable to work/sick leave	70 (3)	39 (4)	
Retired	643 (24)	243 (22)	
Student	190 (7)	74 (7)	
Homemaker	42 (2)	23 (2)	
Missing (%)	166 (6)	191 (18)	
Pre-injury health status			
Pre-injury ASA-PS classification			< .001
Normal healthy patient	1527 (57)	592 (57)	
Mild systemic disease	872 (33)	334 (30)	
Severe systemic disease	233 (9)	115 (11)	
Missing (%)	34 (1)	56 (5)	
History of substance abuse^c^			< .001
Yes	72 (3)	58 (5)	
Missing (%)	43 (2)	59 (5)	
Pre-injury mental health problems^d^			< .001
Yes	268 (10)	124 (11)	
Missing (%)	43 (2)	59 (5)	
Injury characteristics			
Cause of Injury			< .001
Road traffic accident	1041 (39)	371 (34)	
Incidental fall	1187 (45)	486 (44)	
Other non-intentional injury	239 (9)	84 (8)	
Violence or assault	125 (5)	99 (9)	
Suicide attempt	22 (1)	13 (1)	
Missing (%)	52 (2)	44 (4)	
GCS (3–15)			< .001
Mild (13–15)	1981 (74)	713 (65)	
Moderate/Severe (3–12)	605 (23)	338 (31)	
Missing	80 (3)	46 (4)	
ISS (0–75) (Median, [IQR])	13 [8–25]	16 [9–28]	< .001
Missing (%)	34 (1)	17 (1)	
Intracranial traumatic abnormalities (present)	1381 (52)	555 (51)	
Missing (%)	168 (6)	132 (12)	
MEI^e^			> .05
Yes	909 (34)	410 (37)	
Total percentage of observations of baseline characteristics missing	3	7	
Mental health problems 2 weeks post injury (*N* = 609)			
Depression (0–27)	5 [1–10]	NA	
Missing (%)	77 (2054)		
Anxiety (0–21)	2 [0–6]	NA	
Missing (%)	77 (2054)		
Post-traumatic stress disorder (0–72)	9 [3–19]	NA	
Missing (%)	77 (2057)		

*AIS* Abbreviated Injury Scale, *ASA-PS* The American Society of Anesthesiologists-physical status classification system, *GCS* Glasgow Coma Scale, *ISS* Injury Severity Score, *N* number, *MEI* major extracranial injury, *SD* standard deviation

*p-values from ANOVA for continuous and *χ*^2^ statistics for categorical variables

^a^Patients < 18 years of age (*N* = 158) and non-responders (*N* = 1588) were excluded

^b^Patients < 18 years of age (*N* = 108) and deceased patients (*N* = 491) were excluded

^c^Patients with a history of substance abuse disorder prior to the injury

^d^Patients with a history of anxiety, depression, sleep disorders, or schizophrenia prior to the injury

^e^Patients with an Abbreviated Injury Scale ≥ 3 regarding the following body regions; face, cervical spine, thorax/chest, abdomen/pelvic contents, extremities and pelvic girdle, or external (skin), thus excluding head and neck

Responders and non-responders showed significant differences regarding baseline characteristics (Table 1). Non-responders had a higher median age (47 vs. 51 years), and were more often male (71 vs. 65%) (Table 1). Furthermore, they were more frequently diagnosed with moderate and severe TBI than responders, and had higher median injury severity score (16 vs. 13).

**Fig. 1:**
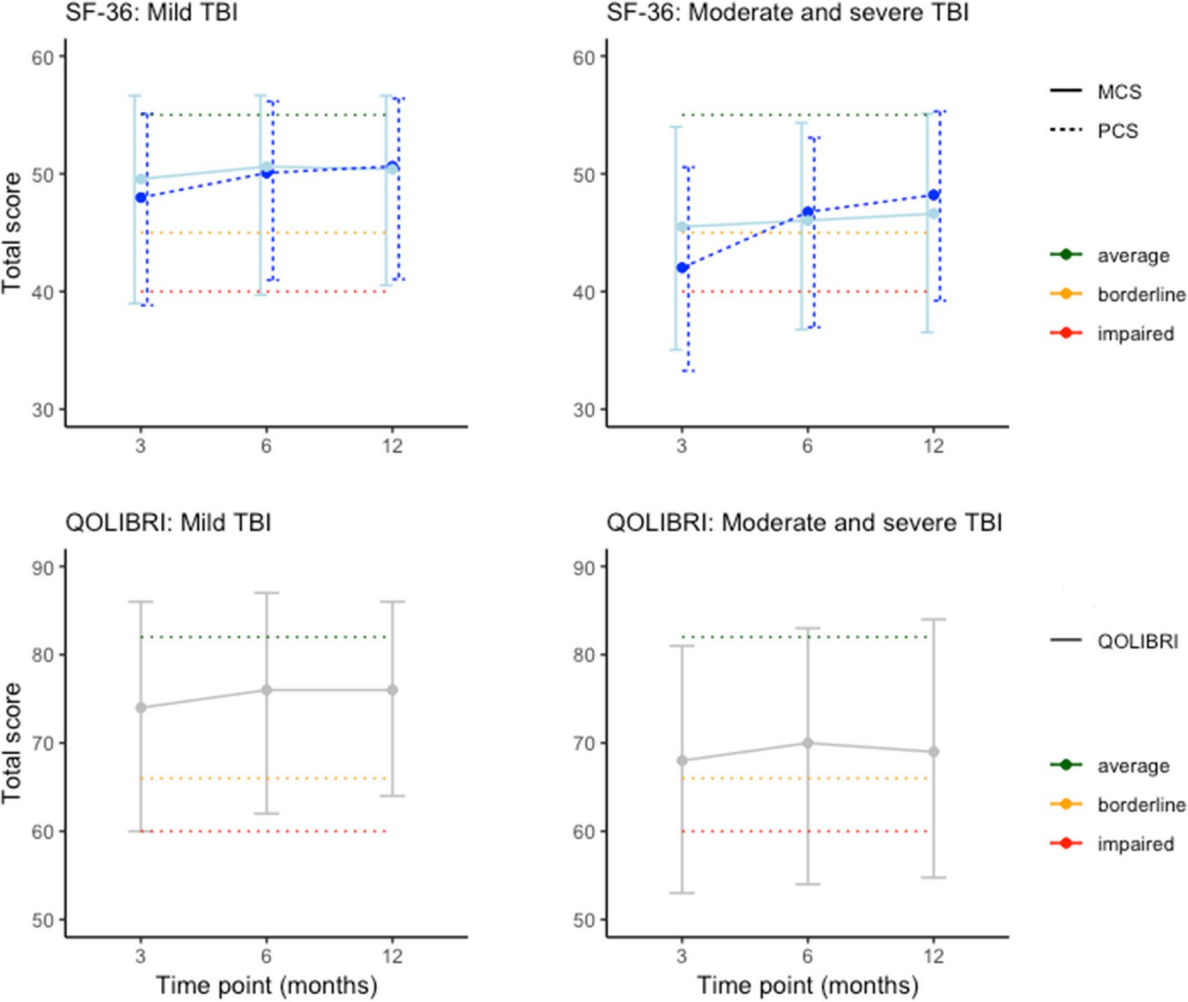
Plots of the median SF-36v2 physical and mental health component summary scores (top) and the Quality of Life after Traumatic Brain Injury (bottom) by time point for mild (left), and moderate and severe TBI (right). For the SF-36v2, scores of 45–55 are considered within the average range (green/upper dotted line), scores of 40–45 are considered borderline (orange/middle dotted line), and scores below 40 (red/lower dotted line) are considered impaired (Ware et al. 2007). For the QOLIBRI, scores of 67–82 are considered within the average range (green/upper dotted line), scores of 60–66 are considered borderline (orange/middle dotted line), and scores below 60 (red/lower dotted line) are considered impaired (Wilson et al. 2017). (Color figure online)

The median PCS, MCS and QOLIBRI total scores increased between 3 and 12 months post injury. The largest improvements were observed between 3 and 6 months (Fig. 1; Supplementary Table 1). PCS showed larger improvements than MCS in patients after mild as well as patients after moderate and severe TBI. At 6 months, 23% of patients after mild and 33% of patients after  moderate and severe TBI fell within the ‘impaired’ category on the PCS. On the MCS, 26% of patients after mild and 33% of patients after moderate and severe TBI had impaired HRQoL, and on the QOLIBRI 22% of patients after mild and 34% of patients after moderate and severe TBI classified as ‘impaired’ 6 months post injury. As expected, patients after moderate and severe TBI had lower median HRQoL scores than patients after mild TBI at every time point. The MCS and QOLIBRI (spearman 0.73) were more strongly related than with PCS (spearman 0.26 with MCS and 0.57 with QOLIBRI; Supplementary Fig. 2).

### Model development

For the predictor values most (97%) observations were complete. Of the predictors with the highest percentage missing, 89% and 94% of observations were complete (Table 1).

#### Physical health component summary score

**Fig. 2:**
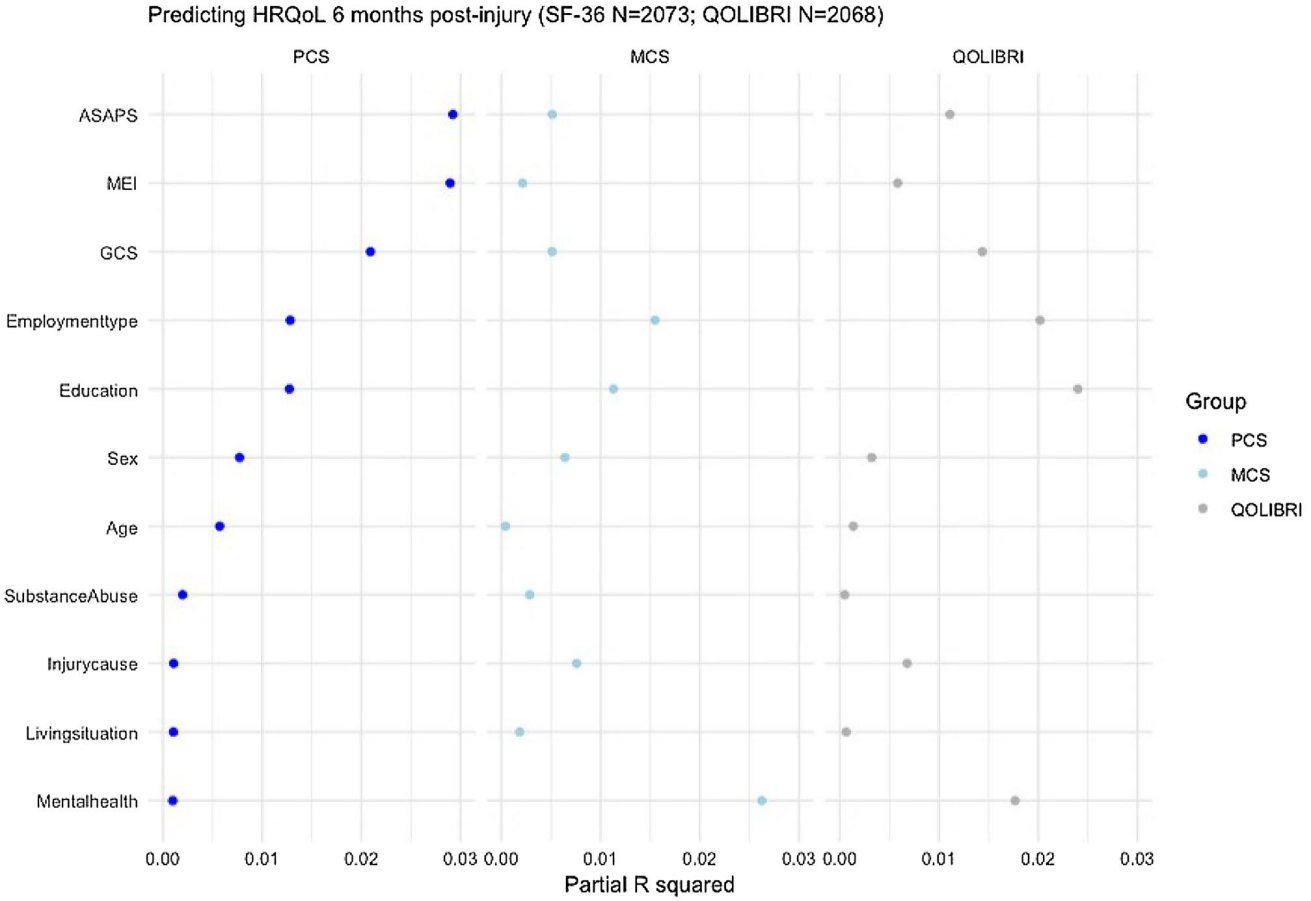
Contribution of predictors to partial explained variance (*R*^2^) of the models for PCS (left), MCS (middle), and QOLIBRI (right). The partial *R*^2^ is calculated as follows: Total *R*^2^ of multivariable model − *R*^2^ multivariable model without individual predictor/Total *R*^2^ of multivariable model without individual predictor = Partial *R*^2^

The strongest predictors of PCS 6 months after TBI were GCS, MEI, and pre-injury health status (ASA-PS) (Table 2; Fig. 2). We found no significant interactions between GCS and the other candidate predictors (*p* > 0.05), indicating that predictors of PCS did not differ between patients with mild (GCS ≥ 13), and moderate and severe TBI (GCS ≤ 12). Severe systemic disease had a strong prognostic effect, indicating that patients with severe pre-injury comorbidities had lower PCS 6 months post injury (Table 2; Supplementary Fig. 3). The model had an *R*^2^ of 11% when the three strongest predictors were considered in the core model. The extended model, also including age, sex, type of employment, and level of education, performed notably better (*R*^2^ = 19%).

**Table 2:** Regression coefficients and 95% confidence intervals for the SF-36v2 physical health component summary score (PCS) with multivariable linear regression analysis

PCS	Core Model	Extended Model	Full Model
Constant	46	49	49
Predictors			
GCS	0.35 (0.25, 0.46)	0.38 (0.28, 0.49)	0.39 (0.28, 0.49)
MEI (No^a^)			
Yes	− 3.7 (− 4.6, − 2.8)	− 4.2 (− 5.1, − 3.3)	− 4.1 (− 5.0, − 3.1)
ASA-PS (Healthy patient^a^)			
Mild systemic disease	− 4.0 (− 5.0, − 3.1)	− 2.0 (− 3.0, − 1.0)	− 2.0 (− 3.0, − 0.96)
Severe systemic disease	− 10.0 (− 12.0, − 8.9)	− 7.2 (− 8.8, − 5.5)	− 7.3 (− 9.0, − 5.7)
Education (College/Uni degree^a^)			
Currently in school		− 1.7 (− 2.9, − 0.51)	− 1.8 (− 3.0, − 0.60)
None/Primary school		− 4.3 (− 5.8, − 2.8)	− 4.3 (− 5.8, − 2.8)
Secondary/high school		− 1.5 (− 2.6, − 0.38)	− 1.6 (− 2.7, − 0.45)
Employment (Working^a^)			
Homemaker		− 4.4 (− 8.2, − 0.55)	− 4.6 (− 8.5, − 0.81)
Student		0.41 (− 1.4, 2.2)	0.45 (− 1.4, 2.3)
Retired		− 1.3 (− 2.7, 0.10)	− 1.4 (− 2.8, − 0.06)
Unable to work/sick leave		− 6.3 (− 8.8, − 3.7)	− 6.1 (− 8.8, − 3.5)
Unemployed		− 3.2 (− 5.1, − 1.2)	− 3.0 (− 5.0, − 1.0)
Age (per decade)		− 0.73 (− 1.0, − 0.36)	− 0.74 (− 1.1, − 0.36)
Sex (Male^a^)			
Female		− 2.1 (− 3.0, − 1.2)	− 2.0 (− 2.9, − 1.1)
Injury cause (Road traffic^a^)			
Incidental fall			0.71 (− 0.24, 1.7)
Other non-intentional injury			− 0.50 (− 1.1, 2.1)
Violence/Assault			− 0.18 (− 2.0, 2.3)
Suicide attempt			− 1.4 (− 5.8, 2.9)
Pre-injury substance abuse (No^a^)			
Yes			3.2 (0.43, 6.0)
Pre-injury mental health problems (No^a^)			
Yes			− 1.2 (− 2.6, 0.26)
Living arrangement (Together^a^)			
Alone			− 0.87 (− 1.9, 0.16)
*R*^2^ development cohort	0.13	0.20	0.21
Optimism	0.01^b^	0.01	0.02
*R*^2^ after bootstrap validation	–	0.19	0.19

Model performance indicated by explained variance (*R*^2^) and bootstrap validation for each model (*N* = 2073)

^a^Reference category of categorical variable

^b^Optimism of the core model is estimated to be similar to that of the extended model

*Core model* Glasgow Coma Scale, Major extracranial injury and pre-injury health status (ASA-PS), *Extended model* core plus education, employment, age and sex, *Full model* extended plus injury cause, pre-injury substance abuse, pre-injury mental health problems, and living arrangement

#### Mental health component summary score

The strongest predictors of MCS 6 months after TBI were pre-injury mental health problems, level of education, and type of employment (Table 3; Fig. 2). Again, we found no significant interactions between GCS and the other candidate predictors (*p* > 0.05). Patients with a low level of education, as well as those who are unemployed, unable to work, or homemakers had lower MCS 6 months after injury (Table 3; Supplementary Fig. 3). The model had an *R*^2^ of 6% when the three strongest predictors, pre-injury mental health problems, level of education and type of employment, were considered in the core model. The extended model, also including age, employment, education and sex, performed somewhat better (*R*^2^ = 9%).

**Table 3:** Regression coefficients and 95% confidence intervals for the SF-36v2 mental health component summary score (MCS) with multivariable linear regression analysis

MCS	Core Model	Extended Model	Full Model
Constant	49	45	44
Predictors			
Pre-injury mental health problems (No^a^)			
Yes	− 7.5 (− 9.2, − 5.9)	− 6.9 (− 8.6, − 5.1)	− 6.8 (− 8.6, − 5.1)
Education (College/Uni degree^a^)			
Currently in school	− 1.7 (− 3.2, − 0.28)	− 1.8 (− 3.3, − 0.40)	− 1.8 (− 3.3, − 0.39)
None/Primary school	− 4.4 (− 6.1, − 2.6)	− 4.3 (− 6.1, − 2.6)	− 4.4 (− 6.1, − 2.6)
Secondary/high school	− 0.96 (− 2.3, 0.36)	− 0.85 (− 2.2, 0.46)	− 0.84 (− 2.1, 0.47)
Employment (Working^a^)			
Homemaker	− 6.4 (− 11.0, − 1.9)	− 4.4 (− 8.9, 0.12)	− 4.5 (− 9.1, 0.07)
Student	− 0.33 (− 2.3, 1.6)	− 0.48 (− 2.4, 1.5)	− 0.31 (− 2.5, 1.9)
Retired	2.1 (0.87, 3.3)	2.5 (1.1, 3.8)	2.3 (0.60, 4.0)
Unable to work/sick leave	− 5.8 (− 8.9, − 2.7)	− 4.5 (− 7.6, − 1.4)	− 4.6 (− 7.7, − 1.5)
Unemployed	− 4.1 (− 6.5, − 1.7)	− 4.0 (− 6.4, − 1.6)	− 4.0 (− 6.4, − 1.6)
Injury cause (Road traffic^a^)			
Incidental fall		2.2 (1.1, 3.4)	2.2 (1.1, 3.3)
Other non-intentional injury		1.2 (− 0.67, 3.1)	1.2 (− 0.69, 3.1)
Violence or Assault		0.01 (− 2.6, 2.6)	− 0.04 (− 2.5, 2.6)
Suicide attempt		4.9 (− 0.15, 10.0)	4.9 (− 0.15, 10.0)
GCS		0.22 (0.10, 0.34)	0.22 (0.09, 0.34)
ASA-PS (Healthy patient^a^)			
Mild systemic disease		− 0.95 (− 2.1, 0.21)	− 1.0 (− 2.2, 0.20)
Severe systemic disease		− 3.4 (− 5.4, − 1.5)	− 3.5 (− 5.5, − 1.5)
Pre-injury substance abuse (No^a^)			
Yes		− 4.4 (− 7.7, − 1.1)	− 4.3 (− 7.6, − 1.0)
Sex (Male^a^)			
Female		− 2.1 (− 3.2, − 1.0)	− 2.1 (− 3.2, − 1.0)
Living arrangement (Together^a^)			
Alone		− 1.3 (− 2.5, 0.06)	− 1.3 (− 2.5, − 0.07)
MEI (No^a^)			
Yes		− 1.2 (− 2.3, − 0.15)	− 1.2 (− 2.3, − 0.15)
Age (per decade)			0.08(− 0.37, 0.53)
*R*^2^ development cohort	0.08	0.11	0.11
*R*^2^ optimism	0.02^b^	0.02	0.02
*R*^2^ after bootstrap validation	–	0.09	0.09

Model performance indicated by explained variance (*R*^2^) and bootstrap validation for each model (*N* = 2073)

*Core model* pre-injury mental health problems, education and employment, *Extended model* core plus injury cause, GCS, ASA-PS, living arrangement, MEI and sex, *Full model* extended plus age

^a^Reference category of categorical variable

^b^Optimism of the core model is estimated to be similar to that of the extended model

#### Quality of Life after Traumatic Brain Injury total score

The strongest predictors of the QOLIBRI total score at 6 months were type of employment, level of education and pre-injury mental health problems (Table 4; Fig. 2), which was similar to the MCS. Again, we found no significant interactions between GCS and the other candidate predictors (p > 0.05). Model performance for the QOLIBRI was intermediate to that of the models for PCS and MCS (*R*^2^ 13%, compared to 18% for PCS and 9% for MCS full models) (Table 4).

**Table 4:** Regression coefficients and 95% confidence intervals for the Quality of Life after Traumatic Brain Injury (QOLIBRI) total score with multivariable linear regression analysis

QOLIBRI	Core Model	Extended Model	Full Model
Constant	78	70	73
Predictors			
Pre-injury mental health problems (No^a^)			
Yes	− 9.8 (− 12.0, − 7.2)	− 9.0 (− 12.0, − 6.3)	− 8.8 (− 11.0, − 6.2)
Education (College/Uni degree^a^)			
Currently in school	− 5.1 (− 7.3, − 2.8)	− 5.0 (− 7.2, − 2.8)	− 5.0 (− 7.2, − 2.8)
None/Primary school	− 11.0 (− 14.0, − 8.0)	− 10.0 (− 13.0, − 7.6)	− 10.0 (− 13.0, − 7.4)
Secondary/high school	− 4.8 (− 6.9, − 2.8)	− 4.4 (− 6.4, − 2.4)	− 4.5 (− 6.5, − 2.5)
Employment (Working^a^)			
Homemaker	− 12.0 (− 19.0, − 5.6)	− 10.0 (− 17.0, − 3.1)	− 9.1 (− 16.0, − 2.2)
Student	− 1.6 (− 1.5, 4.6)	− 1.3 (− 1.7, 4.3)	− 0.11 (− 3.5, 3.2)
Retired	− 0.30 (− 2.2, 1.6)	− 0.47 (− 1.6, 2.5)	2.0 (− 0.62, 4.6)
Unable to work/sick leave	− 11.0 (− 16.0, − 6.4)	− 9.4 (− 14.0, − 4.8)	− 8.6 (− 13.0, − 3.9)
Unemployed	− 9.4 (− 13.0, − 5.7)	− 9.1 (− 13.0, − 5.4)	− 9.2 (− 13.0, − 5.5)
Injury cause (Road traffic^a^)			
Incidental fall		2.8 (1.1, 4.6)	3.1 (1.4, 4.9)
Other non-intentional injury		3.2 (0.32, 6.0)	3.3 (0.43, 6.1)
Violence or Assault		− 1.0 (− 5.0, 3.0)	− 1.2 (− 5.2, 2.8)
Suicide attempt		3.1 (− 4.8, 11.0)	3.2 (− 4.7, 11.0)
GCS		0.56 (0.37, 0.74)	0.57 (0.38, 0.76)
ASA-PS (Healthy patient^a^)			
Mild systemic disease		− 2.4 (− 4.2, − 0.66)	− 1.9 (− 3.8, 0.09)
Severe systemic disease		− 8.9 (− 12.0, − 5.8)	− 8.1 (− 11.0, − 5.0)
Pre-injury substance abuse (No^a^)			
Yes			− 2.9 (− 8.3, 2.4)
Sex (Male^a^)			
Female		2.4 (0.74, 4.0)	− 2.3 (− 4.0, − 0.69)
Living arrangement (Together^a^)			
Alone			− 1.2 (− 3.1, 0.68)
MEI (No^a^)			
Yes		− 3.1 (− 4.8, − 1.4)	− 3.2 (− 4.9, − 1.5)
Age (per decade)		− 0.62 (− 1.3, 0.07)	− 0.63 (− 1.3, 0.06)
*R*^2^ development cohort	0.10	0.15	0.15
*R*^2^ optimism	0.02^b^	0.02	0.02
*R*^2^ after bootstrap validation	–	0.13	0.13

Model performance indicated by explained variance (*R*^2^) and bootstrap validation for each model (*N* = 2068)

*Core model* Education, employment type and pre-injury mental health problems, *Extended model* core plus injury cause, GCS, ASA-PS, sex, MEI, and age, *Full model* extended plus pre-injury substance abuse, and living arrangement

^a^Reference category of categorical variable

^b^Optimism of the core model is estimated to be similar to that of the extended model

#### Early HRQoL assessment, ongoing mental health and intracranial lesions

**Fig. 3:**
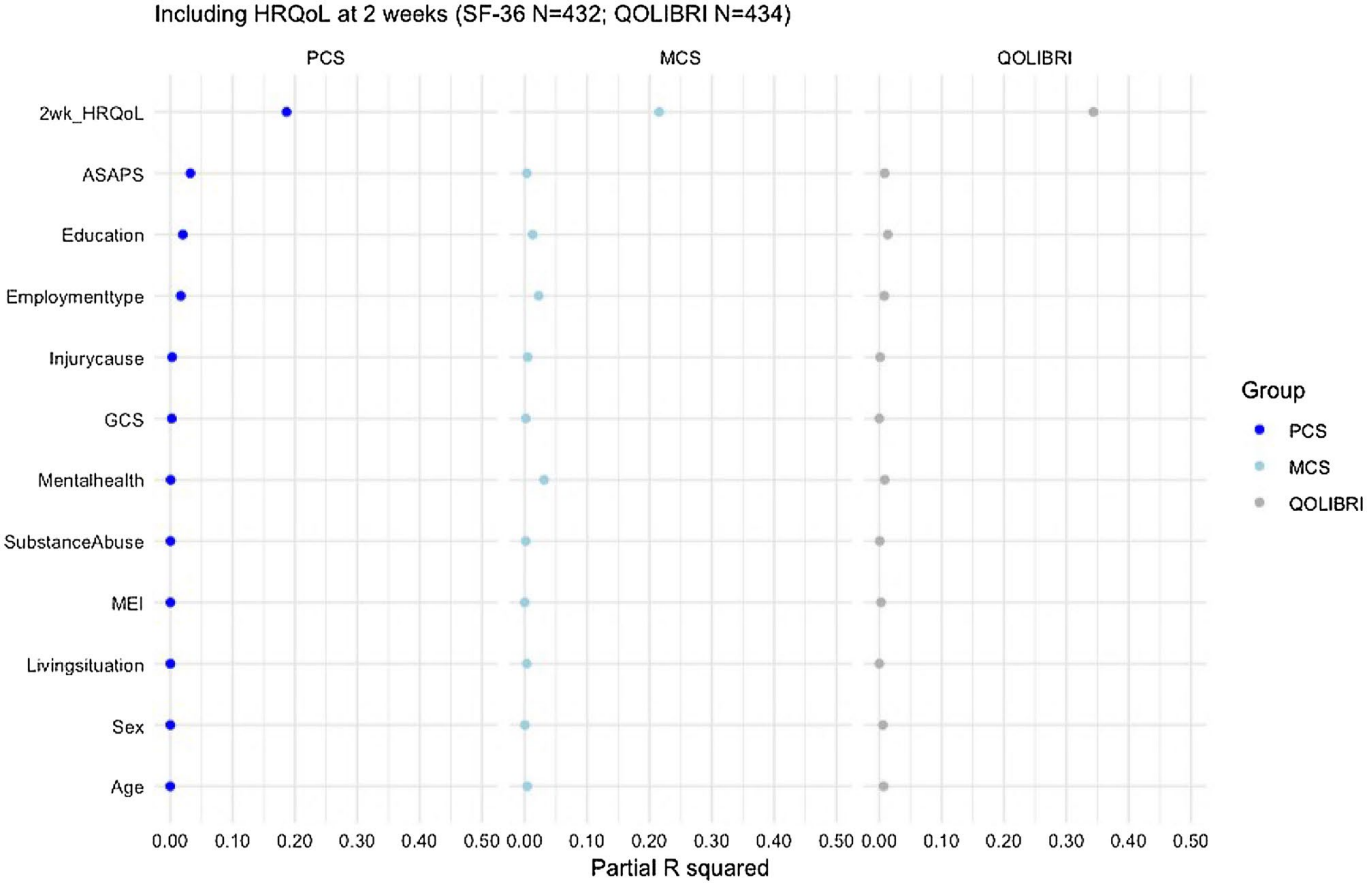
Contribution of predictors to partial explained variance (*R*^2^) of the full models for PCS (left), MCS (middle), and QOLIBRI (right) including early HRQoL assessment at 2 weeks

In a subgroup of patients following predominantly mild TBI (99%), early HRQoL assessment at 2 weeks (SF-36v2 *N* = 432 and QOLIBRI *N* = 434) had substantial incremental value (PCS *R*^2^ 37% compared to 15% of the full model without 2 week PCS; MCS 36% compared to 12% of the full model without 2 week MCS; QOLIBRI 48% compared to 10% of the full model without 2 week QOLIBRI) (Fig. 3). Similarly, depression, anxiety, and PTSD at 2 weeks (SF-36v2 *N* = 418 and QOLIBRI *N* = 420) had substantial incremental value for the prediction of MCS and the QOLIBRI (MCS *R*^2^ = 35% compared to 11% of the full model without 2 week depression, anxiety and PTSD; QOLIBRI = 37% compared to 12% of the full model without 2 week depression, anxiety and PTSD). However, the addition of mental health problems 2 weeks post injury had limited incremental value for the prediction of PCS (PCS *R*^2^ = 22% compared to 16% of the full model without 2 week depression, anxiety and PTSD). Furthermore, for the prediction of PCS, MCS and the QOLIBRI, the addition of intracranial traumatic abnormalities (*N* = 1642 and 1639) had no or limited incremental value (PCS *R*^2^ = 20% compared to 19% of the full model without intracranial traumatic abnormalities; MCS 10% compared to 10%; QOLIBRI 14% compared to 13%).

#### Sensitivity analyses

Model performance was similar when proxy responses (PCS and MCS *N* = 98, QOLIBRI *N* = 93) were excluded. The full models also performed similarly when 3 month rather than 6 month HRQoL was predicted (PCS *R*^2^ 20% vs 19% when the model was fitted for 6 month outcome, respectively; MCS *R*^2^ 9% vs 9%; QOLIBRI *R*^2^ 14% vs 13%;). Furthermore, the models performed similarly when missing 6 month HRQoL outcomes (*N* = 462) were imputed for with HRQoL outcomes on 3 and 12 months (PCS *R*^2^ 20% vs 19%, respectively; MCS *R*^2^ 9% vs 9%; QOLIBRI *R*^2^ 13% vs 13%) (Supplementary Tables 2, 3, 4). As expected, the predictive value of GCS diminished when patients were separated based on GCS (Mild ≥ 13, Moderate and Severe ≤ 12) (Supplementary Fig. 4). The models were fitted for impaired PCS and MCS (< 40) and QOLIBRI total scores (< 60). The strongest predictors of impaired PCS were GCS, pre-injury health status and MEI (Supplementary Table 5). For impaired MCS the strongest predictors were pre-injury mental health problems, employment type and level of education (Supplementary Table 6). The strongest predictors of impaired QOLIBRI total score were GCS, level of education, and employment type (Supplementary Table 7).

#### Model presentation

The proposed models were presented with nomograms (Supplementary Figs. 5, 6, 7). Prognostic HRQoL scores at 6 months post injury can be calculated for individual patients using the formulas (Textbox 1; Supplementary Table 8).

**Textbox 1:** Example of calculation of individual SF-36v2 physical (PCS) and mental (MCS) component summary scores and the Quality of Life after Traumatic Brain Injury (QOLIBRI) total score at 6 months post injury based on the core models

	Patient characteristics	PCS score (*T*-scores)	MCS score (*T*-scores)	QOLIBRI score (0–100)
Constant		46	49	78
GCS	13	0.35 × 13		
MEI	Yes	− 3.7 × 1		
ASA-PS	Mild systemic disease	− 4.0 × 1		
Pre-injury mental health problems	Yes		− 7.5 × 1	− 9.8 × 1
Education level	High school		− 0.96 × 1	− 4.8 × 1
Employment type	Retired		2.1 × 1	− 0.30 × 1
Sum score		43	43	63

## Discussion

We developed simple and more extended models for predicting Health-Related Quality of Life (HRQoL) 6 months after traumatic brain injury (TBI), separately for the SF-36v2 physical (PCS) and mental component summary scores (MCS) and the Quality of Life after Traumatic Brain Injury (QOLIBRI) total score. Medical and injury-related characteristics were most important for the prediction of PCS, whereas patient-related characteristics were more relevant for prediction of MCS and the QOLIBRI. Moderate model performance is indicative for the complexity of predicting HRQoL. Substantial improvement in model performance was achieved by including 2-week HRQoL assessment.

Although previously indicated predictors of HRQoL following TBI were also relevant in our study the proportion explained variance (*R*^2^) of the models was moderate. Models that include predictors that move beyond baseline assessment, also known as dynamic or longitudinal predictors, have been proposed to update existing models and potentially improve performance [21]. Prior studies have shown the importance of aspects of current status, including emotional state, for the prediction of HRQoL following TBI [14, 38, 43]. As expected, our study demonstrated that early HRQoL assessment substantially improved model performance in a subset of patients with predominantly mild TBI; the *R*^2^ for PCS was 38% compared to 17% of the full model without 2 week HRQoL; for MCS the increase was to 35% from 12%, and for the QOLIBRI the *R*^2^ increased from 19 to 54%. In our study, HRQoL was highly variable between TBI patients over time, whereas within patients HRQoL scores might be more stable. This could explain the substantial incremental value of 2 week HRQoL for the prediction of 6 month HRQoL outcomes. In our study, adherence varied across time points; 2 week HRQoL assessment was only available in patients that were seen in the Emergency Room (ER) and discharged or in the hospital ward other than the ICU, which almost exclusively comprised mild TBI patients (99%) without MEI (91%). Therefore, the incremental value of early HRQoL assessment can only be generalized to patients following mild TBI. Early after injury, patients might be unable or less inclined to respond to questionnaires. Although patient-reported outcomes are increasingly reported in clinical practice, variable or low adherence over time makes early follow-up assessments less feasible to collect, which limits the clinical applicability of dynamic prediction models using patient-reported outcomes or assessments. Other longitudinal predictors that can be considered to be included for the prediction of HRQoL following TBI that may be less dependent on patient response are, for instance, biomarkers, duration of hospital stay, and length of coma.

In our study, most patients (74%) classified as mild TBI. More than half (1381/2666, 52%) had intracranial traumatic abnormalities on the initial computed tomography (CT) scan, which might be related to worse long-term outcome and lower HRQoL. In patients following mild TBI, the presence or absence of intracranial traumatic abnormalities is used to differentiate between complicated and uncomplicated mild TBI [44]. A recent study found that although patients after complicated mild TBI reported slightly more post-concussion symptoms compared to those after uncomplicated mild TBI, an abnormality on initial CT was only a weak indicator of these problems after adjusting for baseline covariates (e.g., age, gender, GCS) [45]. However, the relationship between intracranial traumatic abnormalities and HRQoL following TBI has not been examined yet. Our study indicates that when adjusting for patient- and injury-related characteristics, intracranial traumatic abnormalities had limited to no incremental value for the prediction of HRQoL following TBI. As intracranial traumatic abnormalities are relevant to address the heterogeneity in patients following mild TBI [44], a formal investigation of the relationship between intracranial traumatic abnormalities and HRQoL in a subgroup of patients following mild TBI is warranted. A recent study indicates that the Helsinki CT classification was associated with QoL up to 4 years after TBI [46]. Besides the presence of intracranial traumatic abnormalities, more detailed information such as CT lesion phenotypes, their location, extent and clustering could therefore be considered.

TBI can lead to long-term impairments in functional, physical, mental, cognitive, and social domains. Although median MCS was initially higher than PCS at 3 months, PCS showed greater improvements between 6 and 12 months post injury. This indicates that over time mental health was more strongly affected by TBI. These findings also advocate for a multidimensional outcome assessment of TBI that captures a broad range of difficulties patients may experience, including physical, psychosocial and emotional outcomes. Furthermore, prior studies have shown that patients who sustained TBI, on average, show large HRQoL deficits from full recovery after 12 months when measured by population norms [6]. In our study, post hoc analyses confirmed these findings in mild as well as moderate and severe TBI patients; at 12 months 22% of mild and 27% of moderate and severe TBI patients had impaired PCS scores. Similarly, 24% of mild and 35% of moderate and severe TBI patients had impaired MCS scores, and 21% of mild and 33% of moderate and severe TBI patients had impaired QOLIBRI scores at 12 months. This indicates that a subgroup of patients may experience physical and mental limitations one year after TBI. The pattern of HRQoL scores described in our study also indicates a ceiling effect, which is a prominent issue in TBI outcome studies [4].

The strongest predictors of the MCS were pre-injury mental health, level of education, and employment. Based on our findings, we can conclude that patient-related characteristics are more important for the prediction of MCS than injury-related characteristics, such as GCS. In other words, patients’ wellbeing following TBI is more strongly influenced by psychosocial factors than the severity of injury. Furthermore, predictors of functional outcomes differ for patients with mild versus moderate and severe TBI, motivating the development of separate models for these patients [21]. It has been suggested that following moderate and severe TBI, functional outcome is determined by what “the injury brings to the patient”, whereas in mild TBI it is determined by what “the patient brings to the injury” [23]. In contrast, predictors of HRQoL did not significantly differ between patients with mild, and moderate and severe TBI. This might be explained by the fact that HRQoL captures the patient’s subjective experience of their wellbeing in daily life, and is therefore likely to be affected by psychological factors and emotional adjustment. Consequently, patient-related characteristics (e.g., pre-injury mental health, level of education, and employment) were expected to influence HRQoL and predictor effects to vary less by injury severity.

The combined rate of pre-injury mental health problems (Anxiety, depression, sleep disorders, and schizophrenia) was 10%, which is somewhat lower than pre-injury mental health problems of 19% and 13% for anxiety and depression based on structured diagnostic interviews) (Scholten 2016). Between studies, there is a wide variation in prevalence rates of pre-injury anxiety and depressive disorders. This can be explained by differences in study design, patients characteristics, definitions, assessment methods, and measures used to assess psychiatric outcomes.

The models for PCS performed better than those predicting MCS and the QOLIBRI total score (*R*^2^ 19% compared to 9% and 13% of the full models for MCS and QOLIBRI). Patients’ resilience, coping strategies and social support are associated with psychological outcome following TBI [47–50]. Although these psychological processes are typically not assessed in RCTs or observational studies in TBI they have the potential to improve model performance and provide opportunities for focused interventions to improve long-term psychological outcome following TBI. In patients following mild TBI, post-concussion symptoms, relating to a subset of somatic, cognitive, behavioral and emotional symptoms, are negatively associated with HRQoL [51]. Furthermore, cognitive impairments are associated with HRQoL following TBI [52]. Future research should therefore focus on the development of dynamic prediction models for HRQoL following TBI, including resilience, social support, coping, cognitive impairments, and early post-concussion symptoms as (longitudinal) predictors.

The models developed in our study include characteristics that were available at admission and 2 weeks post injury. Reliable information about prognosis is of major importance to patients who sustained TBI and their families. For clinicians it would be notoriously difficult, if not impossible, to predict a patient’s subjective experience of their sequelae in daily life. Prediction models for HRQoL following TBI have the potential to support clinicians to identify patients at increased risk of experiencing limitations in their daily life, who could then be followed more closely and receive early interventions to alleviate the burden of injury. Before prediction models can be considered for implementation in clinical practice, external validation is required to evaluate their performance in new settings.

Strengths of this study include the use of a longitudinal, prospective observational cohort study (CENTER-TBI). Consequently, we made use of a standardized collection of data, and a well-described contemporary cohort of patients. Also, the large sample size of the development cohort allowed for reliable predictions. Another strength is the selection of candidate predictors based on literature and expert knowledge, which is preferred over selection based on data, that may increase the risk for overfitting. The predictors can be easily extracted from patients with standardized questionnaires at admission and early after admission, and are available at the time the model is to be used. Furthermore, we used a generic (SF-36v2) and TBI-specific (QOLIBRI) instrument to assess HRQoL. The SF-36 is validated and most widely used in HRQoL studies and in practice [6]. The proposed models for the SF-36v2 scales can be compared to models for other neurological conditions, such as stroke. Prior research indicates that the QOLIBRI provides additional information to the SF-36 [19].

Several limitations of our study have to be considered. First, candidate predictors were based on literature and expert knowledge. However, among studies, participants, definitions of (HR)QoL, instruments, and time points of HRQoL assessment vary widely [6]. Although prior evidence of predictors is therefore limited our study provides insight in predictors of HRQoL following TBI based on multivariable analysis. Second, living arrangement at admission was considered a proxy of social support and therefore included as a predictor. Social support is associated with psychological outcomes after TBI [50], but it is typically unmeasured in longitudinal studies. Living arrangement might be related to social support; however, we cannot generalize our findings to the effect of social support on HRQoL following TBI. Third, in our study, non-responders were more frequently diagnosed with moderate/severe TBI than responders. Patients with more severe injury might be unable to respond to questionnaires over time. Furthermore, the SF-36v2 is not suitable for patients with major cognitive impairment or language difficulties, and thus an important subgroup of patients with profound disability is excluded. In the future, options to further improve adherence rates among TBI patients should be explored. For instance, researchers and clinicians could combine patients’ healthcare facility visits with reminders to fill in questionnaires or electronic reminders via smartphone applications.

## Conclusion

Whereas prognostic models for functional outcome following TBI typically include medical and injury-related characteristics, our results suggest that patient-related characteristics contribute to the prediction of HRQoL following TBI. Prediction models for HRQoL have the potential to inform clinicians and patients and their families about prognosis 6 months after TBI. However, performance of the proposed models was moderate, which reflects the complexity of predicting HRQoL following TBI.

## Supplementary Information

Below is the link to the electronic supplementary material.

Supplementary file1 (TIFF 8219 kb) **Supplementary Fig. 1** Flow diagram of participants

Supplementary file2 (TIFF 8490 kb) **Supplementary Fig. 2** Correlation matrixes for the SF-36v2 physical (PCS) and mental (MCS) component summary score and the Quality of Life after Traumatic Brain Injury (QOLIBRI) total score at 6 months post-injury. The color saturation and seize of the circle indicate the strength of the relationship: more saturated colors and a larger size of the circle indicate a stronger relationship between the variables. The color of the circle also indicates if the relationship between *x* and *y* is positive (shades of blue), or negative (shades of red)

Supplementary file3 (TIFF 8490 kb) **Supplementary Fig. 3 ** Plots of predictor effects, including confidence intervals, that were included in the full models that predict PCS (left), MCS (middle), and the QOLIBRI total score (right) 6 months after traumatic brain injury. *GCS* Glasgow Coma Scale, *Mental health* pre-injury mental health problems, *Schoolgoing* currently in or with diploma/degree oriented program, *ASA-PS Mild* mild systemic disease pre-injury, *ASA-PS Severe* severe systemic disease pre-injury, *Substance abuse* pre-injury substance abuse

Supplementary file4 (TIFF 8490 kb) **Supplementary Fig. 4** Contribution of predictors to partial explained variance (*R*^2^) of the models for PCS (first row), MCS (second row), and the QOLIBRI (third row) separately for mild (GCS ≥ 13) and moderate and severe TBI (GCS ≤ 12)

Supplementary file5 (TIFF 8490 kb) **Supplementary Fig. 5** Nomograms of predictor effects in the core (top) and extended (bottom) models that predict the SF-36v2 physical (PCS) component summary score 6 months after traumatic brain injury

Supplementary file6 (TIFF 8490 kb) **Supplementary Fig. 6** Nomograms of predictor effects in the core (top) and extended (bottom) models that predict SF-36v2 mental (MCS) component summary score 6 months after traumatic brain injury

Supplementary file7 (TIFF 8490 kb) **Supplementary Fig. 7** Nomograms of predictor effects in the core (top) and extended (bottom) models that predict the Quality of Life after Traumatic Brain Injury (QOLIBRI) total score 6 months post-injury

Supplementary file8 (DOCX 13 kb)

Supplementary file9 (DOCX 16 kb)

Supplementary file10 (DOCX 16 kb)

Supplementary file11 (DOCX 16 kb)

Supplementary file12 (DOCX 14 kb)

Supplementary file13 (DOCX 14 kb)

Supplementary file14 (DOCX 14 kb)

Supplementary file15 (DOCX 14 kb)

## Data Availability

CENTER-TBI is committed to data sharing and in particular to responsible further use of the data. Hereto, we have a data sharing statement in place: https://www.center-tbi.eu/data/sharing. The CENTER-TBI dataset is hugely complex, and the CENTER researchers wish to encourage correct and appropriate use of the data; this means that we encourage researchers to contact the CENTER-TBI Team for any research plans and the Data Curation Team for any help in appropriate use of the data, including sharing of scripts. Requests for data access can be submitted online: https://www.center-tbi.eu/data. The complete Manual for data access is also available online: https://www.center-tbi.eu/files/SOP-Manual-DAPR-2402020.pdf.

## Abbreviations

CENTER-TBI: Collaborative European NeuroTrauma Effectiveness Research in Traumatic Brain Injury
GCS: Glasgow Coma Scale
HRQoL: Health-Related Quality of Life
MEI: Major extracranial injury
MCS: Mental component summary score
PCS: Physical component summary score
PTSD: Post-traumatic stress disorder
TBI: Traumatic brain injury
QOLIBRI: Quality of Life after Brain Injury

## Code availability

The variable names and associated data dictionary elements for predictors and outcomes included in our study can be found here: https://center-tbi.incf.org/_60ae5959e840f1032773d145 and variable names and associated data dictionary elements for imaging data can be found here: https://center-tbi.incf.org/_60ae5a61e840f1032773d146. R code is available upon request.
