# Supplementary figures and images for: Development of prognostic models for Health-Related Quality of Life following traumatic brain injury
Source: Qual Life Res. 2021 Jul 30;31(2):451–71. doi: 10.1007/s11136-021-02932-z (PMC8847302; doi:10.1007/s11136-021-02932-z)

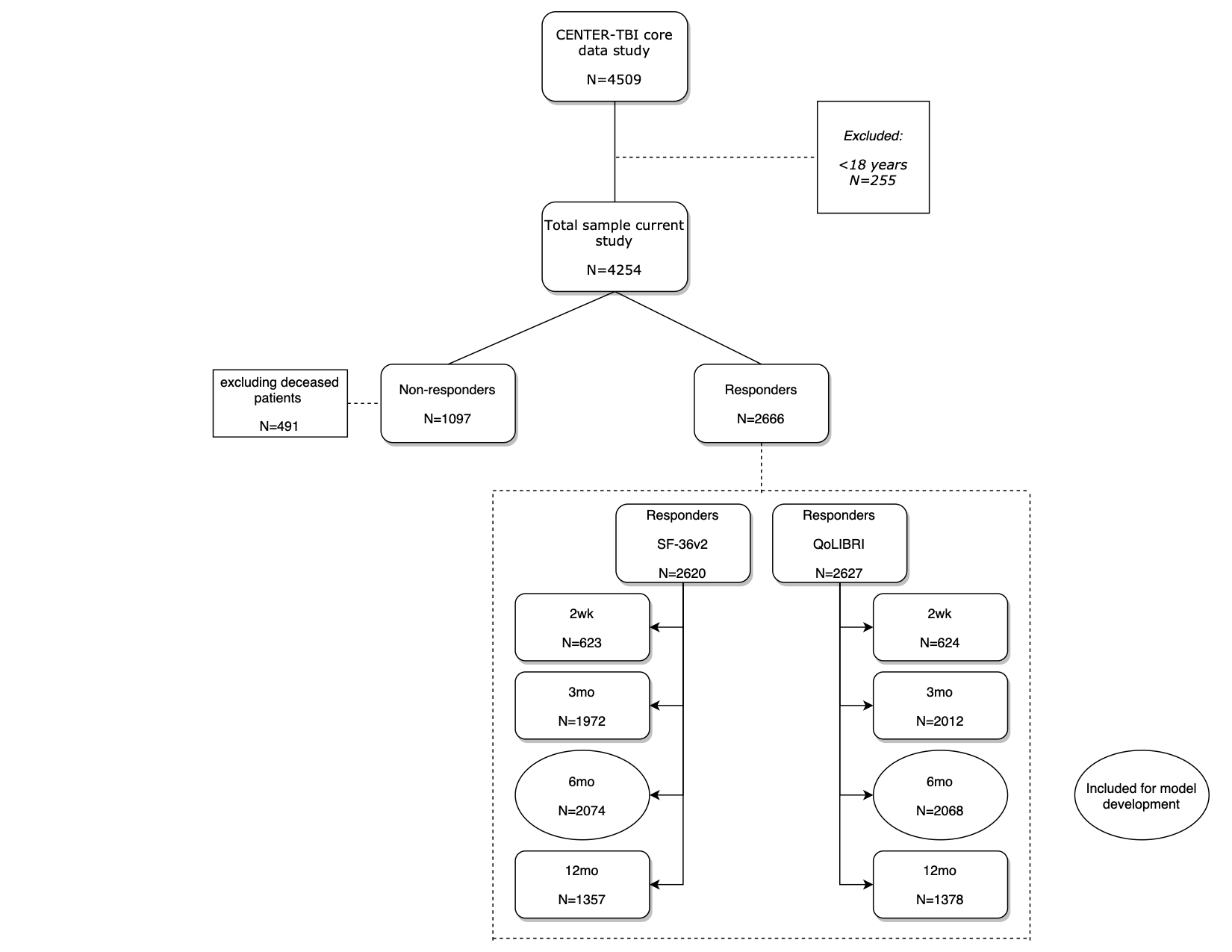

Supplement: Supplementary file 1 — Supplementary file1 (TIFF 8219 kb) Supplementary Fig. 1 Flow diagram of participants [file 11136_2021_2932_MOESM1_ESM.tiff]

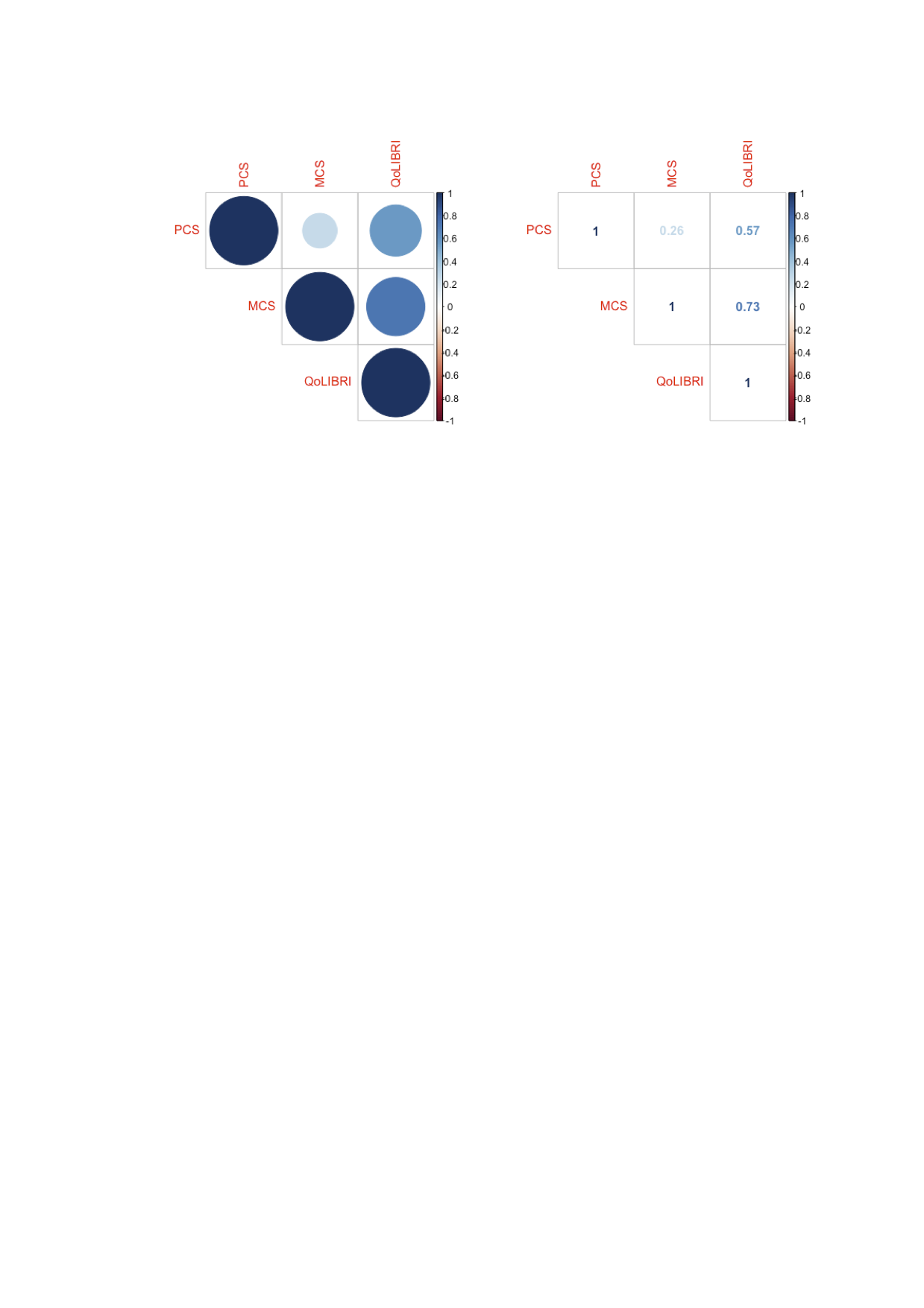

Supplement: Supplementary file 2 — Supplementary file2 (TIFF 8490 kb) Supplementary Fig. 2 Correlation matrixes for the SF-36v2 physical (PCS) and mental (MCS) component summary score and the Quality of Life after Traumatic Brain Injury (QOLIBRI) total score at 6 months post-injury. The color saturation and seize of the circle indicate the strength of the relationship: more saturated colors and a larger size of the circle indicate a stronger relationship between the variables. The color of the circle also indicates if the relationship between x and y is positive (shades of blue), or negative (shades of red) [file 11136_2021_2932_MOESM2_ESM.tiff]

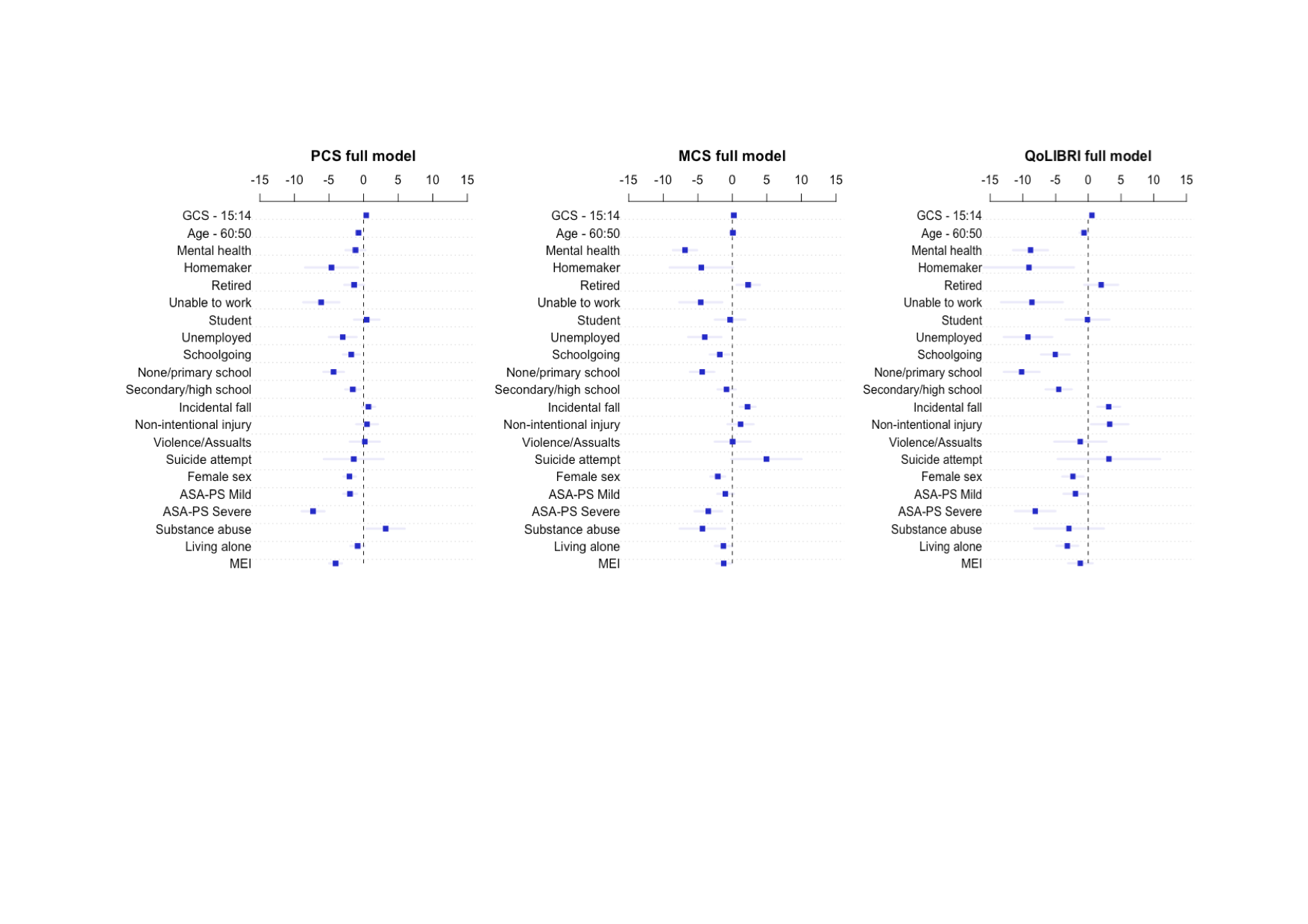

Supplement: Supplementary file 3 — Supplementary file3 (TIFF 8490 kb) Supplementary Fig. 3 Plots of predictor effects, including confidence intervals, that were included in the full models that predict PCS (left), MCS (middle), and the QOLIBRI total score (right) 6 months after traumatic brain injury. GCS Glasgow Coma Scale, Mental health pre-injury mental health problems, Schoolgoing currently in or with diploma/degree oriented program, ASA-PS Mild mild systemic disease pre-injury, ASA-PS Severe severe systemic disease pre-injury, Substance abuse pre-injury substance abuse [file 11136_2021_2932_MOESM3_ESM.tiff]

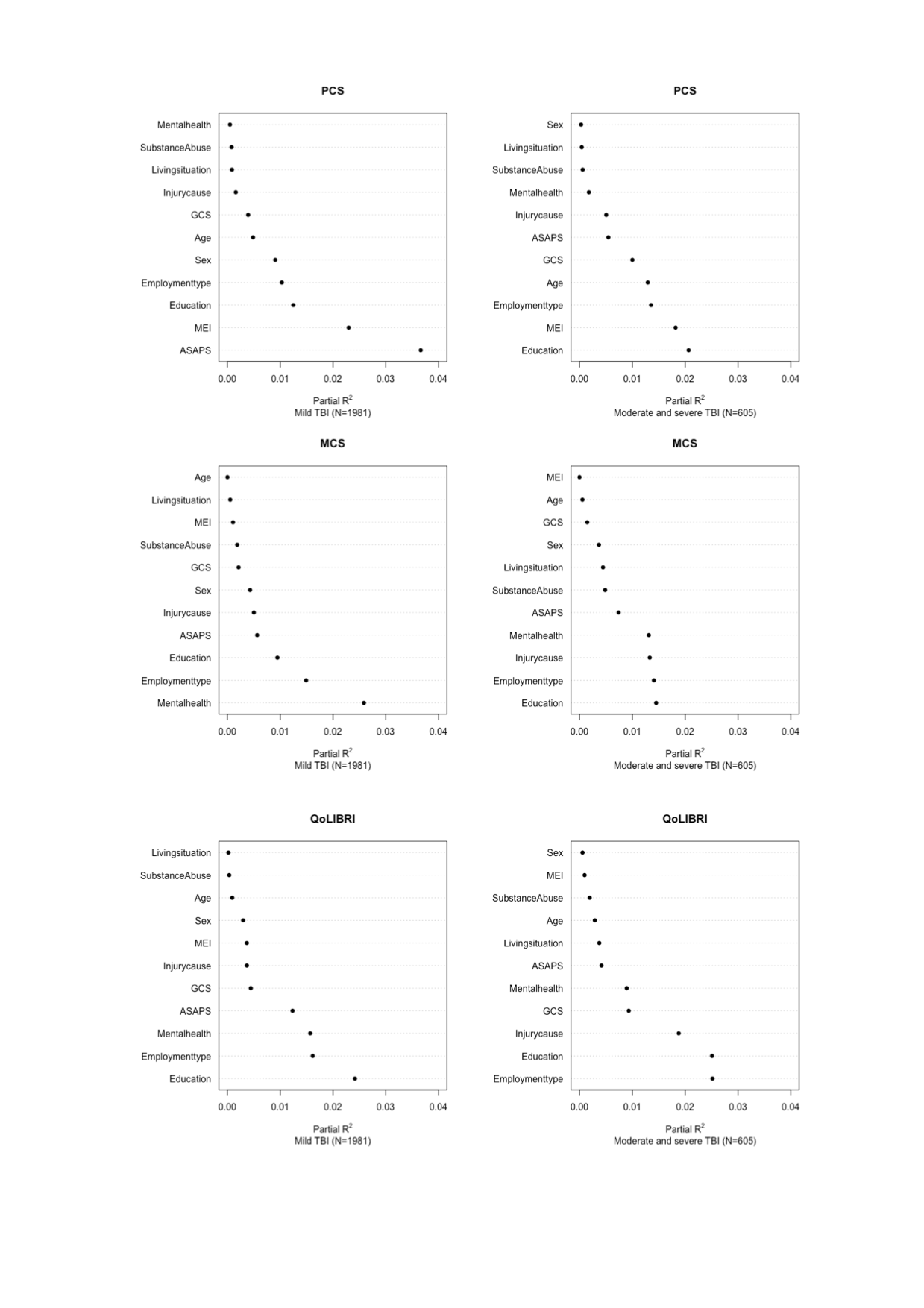

Supplement: Supplementary file 4 — Supplementary file4 (TIFF 8490 kb) Supplementary Fig. 4 Contribution of predictors to partial explained variance (R2) of the models for PCS (first row), MCS (second row), and the QOLIBRI (third row) separately for mild (GCS ≥ 13) and moderate and severe TBI (GCS ≤ 12) [file 11136_2021_2932_MOESM4_ESM.tiff]

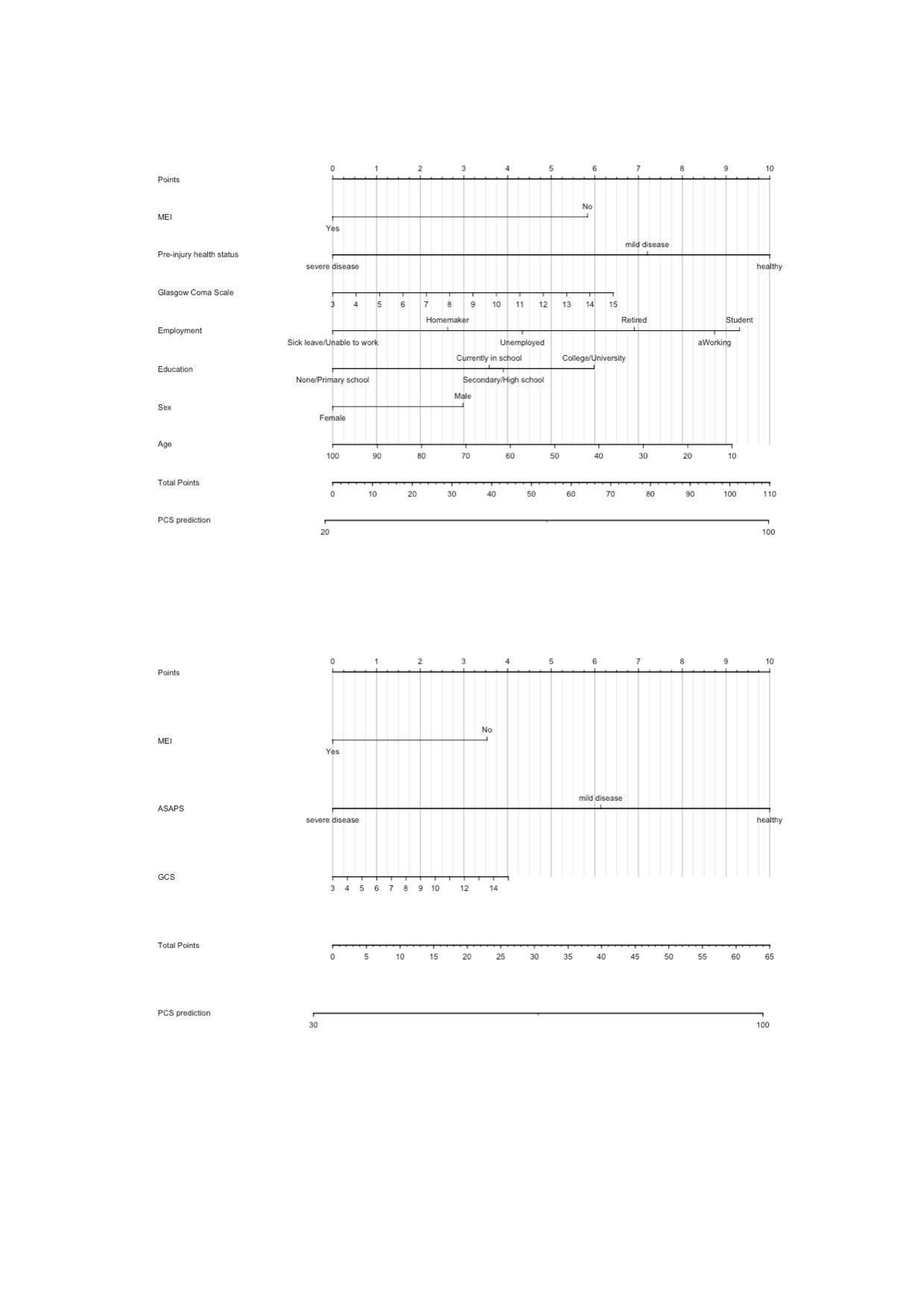

Supplement: Supplementary file 5 — Supplementary file5 (TIFF 8490 kb) Supplementary Fig. 5 Nomograms of predictor effects in the core (top) and extended (bottom) models that predict the SF-36v2 physical (PCS) component summary score 6 months after traumatic brain injury [file 11136_2021_2932_MOESM5_ESM.tiff]

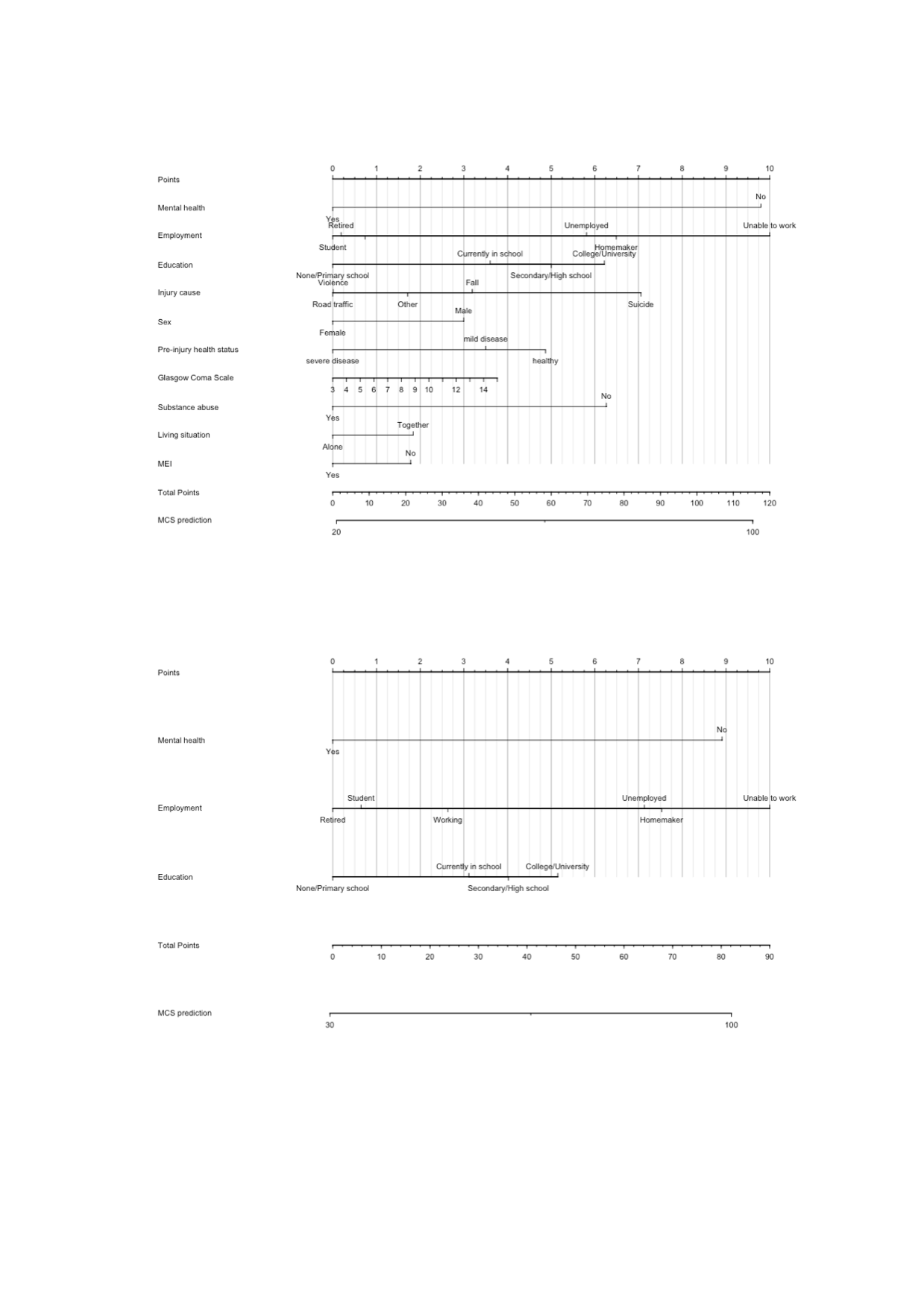

Supplement: Supplementary file 6 — Supplementary file6 (TIFF 8490 kb) Supplementary Fig. 6 Nomograms of predictor effects in the core (top) and extended (bottom) models that predict SF-36v2 mental (MCS) component summary score 6 months after traumatic brain injury [file 11136_2021_2932_MOESM6_ESM.tiff]

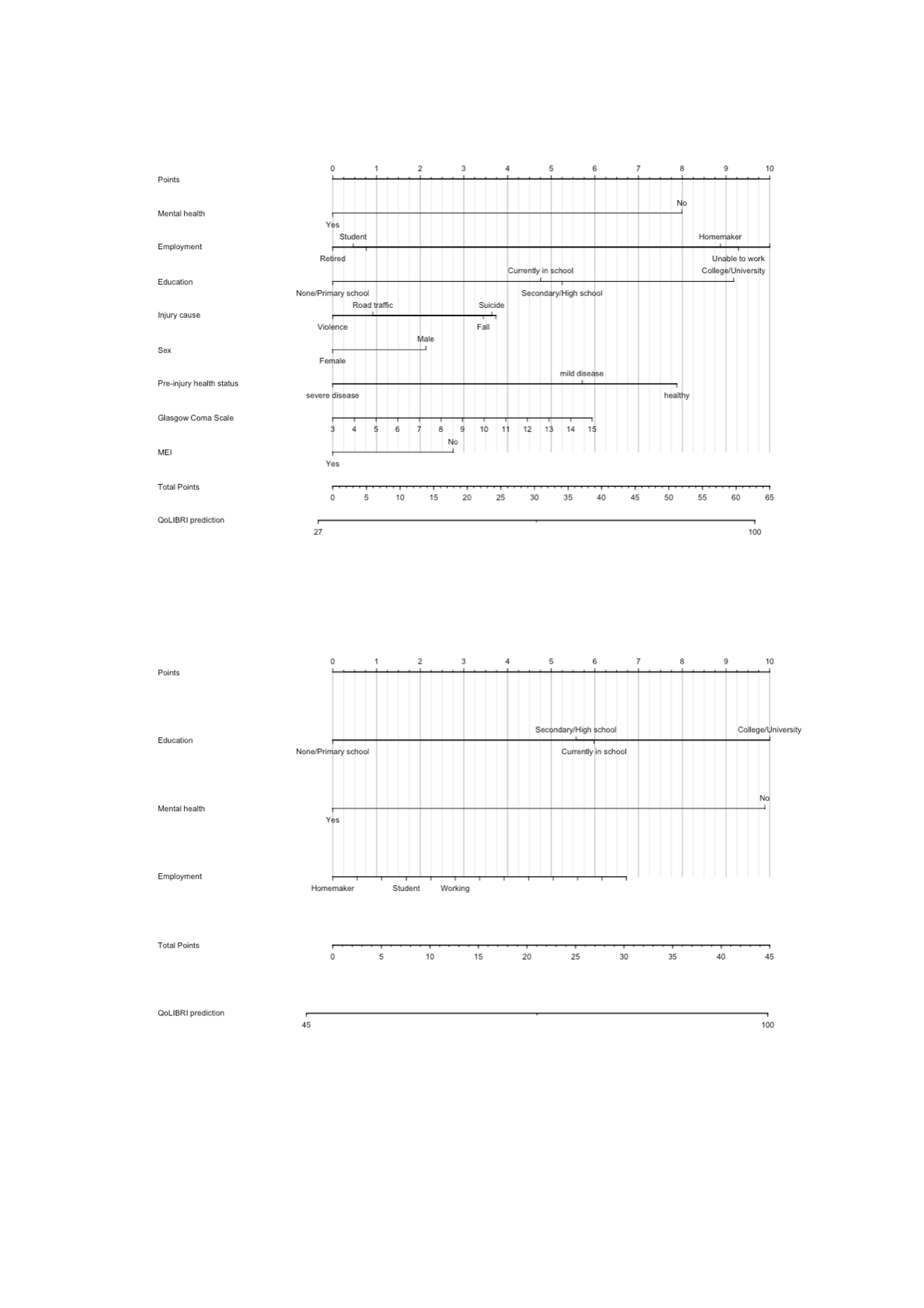

Supplement: Supplementary file 7 — Supplementary file7 (TIFF 8490 kb) Supplementary Fig. 7 Nomograms of predictor effects in the core (top) and extended (bottom) models that predict the Quality of Life after Traumatic Brain Injury (QOLIBRI) total score 6 months post-injury [file 11136_2021_2932_MOESM7_ESM.tiff]
