# Supplementary material for: Development of prognostic models for Health-Related Quality of Life following traumatic brain injury
Source: Qual Life Res. 2021 Jul 30;31(2):451–71. doi: 10.1007/s11136-021-02932-z (PMC8847302; doi:10.1007/s11136-021-02932-z)
Supplement: Supplementary file 8 — Supplementary file8 (DOCX 13 kb) [file 11136_2021_2932_MOESM8_ESM.docx]

| *SF-36v2*  *(N=2620****)*** | **Mild TBI**  **(1981)** | **Moderate and Severe TBI**  **(605)** | **Mild TBI**  **(1981)** | **Moderate and Severe TBI**  **(605)** | **% Missing** |
| --- | --- | --- | --- | --- | --- |
|  | *PCS (Median, [IQR])* | | *MCS (Median, [IQR])* | | |
| **2 weeks** | 43 [35, 52] | 50 [48, 53] | 49 [37, 55] | 45 [37, 50] | 77 |
| **3 months** | 48 [39, 55] | 42 [33, 51] | 50 [39, 57] | 46 [35, 54] | 26 |
| **6 months** | 50 [41, 56] | 47 [37, 53] | 51 [40, 57] | 46 [37, 54] | 22 |
| **12 months** | 51 [41, 56] | 48 [39, 55] | 50 [41, 57] | 47 [37, 55] | 49 |
| *QoLIBRI*  *(N=2627)* | **Mild TBI**  **(1981)** | **Moderate and Severe TBI**  **(605)** |  |  | **% Missing** |
|  |  |  |  |  |  |
| 2 weeks | 72 [58-84] | 84 [77-88] |  |  | 77 |
| 3 months | 74 [60-86] | 68 [53-81] |  |  | 25 |
| 6 months | 76 [62-87] | 70 [54-83] |  |  | 22 |
| 12 months | 76 [64-86] | 69 [55-84] |  |  | 48 |

**Supplementary Table 1** *Patients’* *Health-Related Quality of Life outcomes*
