# Supplementary material for: Development of prognostic models for Health-Related Quality of Life following traumatic brain injury
Source: Qual Life Res. 2021 Jul 30;31(2):451–71. doi: 10.1007/s11136-021-02932-z (PMC8847302; doi:10.1007/s11136-021-02932-z)
Supplement: Supplementary file 9 — Supplementary file9 (DOCX 16 kb) [file 11136_2021_2932_MOESM9_ESM.docx]

**Supplementary Table 2** *Regression coefficients and 95% confidence intervals for the SF-36v2 physical health component summary score (PCS) with multivariable linear regression analysis. Model performance indicated by explained variance (R^2^) and bootstrap validation for each model (N=2535^a^).*

| *PCS* | **Core Model** | **Extended Model** | **Full Model** |
| --- | --- | --- | --- |
| **Constant** | 46 | 49 | 49 |
| **Predictors** |  |  |  |
| **GCS** | 0.36 (0.26, 0.46) | 0.39 ( 0.30, 0.49) | 0.40 (0.30, 0.50) |
| **MEI (No^a^)** |  |  |  |
| **Yes** | -3.6 (-4.5, -2.7) | -4.0 (-4.8, -3.1) | -3.8 (-4.7,-3.0 ) |
| **ASA-PS (Healthy patient^b^)** |  |  |  |
| **Mild systemic disease** | -4.4 (-5.3, -3.5) | -2.2 (-3.3, -1.3) | -2.2 (-3.1,-1.2 ) |
| **Severe systemic disease** | -11.0 (-12.0, -9.3) | -7.4 (-9.0, -5.8) | -7.5 (-9.1,-5.9 ) |
| **Education (College/Uni degree^b^)** |  |  |  |
| **Currently in school** |  | -1.5 (-2.6,-0.33 ) | -1.6 (-2.7,-0.40 ) |
| **None/Primary school** |  | -4.8 (-6.2,-3.4 ) | -4.8 (-6.2,-3.4 ) |
| **Secondary/high school** |  | -1.6 (-2.7, -0.60) | -1.7 (-2.7,-0.65 ) |
| **Employment (Working^b^)** |  |  |  |
| **Homemaker** |  | -4.0 (-7.4,-0.58) | -4.1 (-7.6,-0.71 ) |
| **Student** |  | 0.31 (-1.4, 2.0) | 0.29 (-1.4, 2.0 ) |
| **Retired** |  | -1.4 (-2.7, 0.02) | -1.4 (-2.7,-0.04 ) |
| **Unable to work/sick leave** |  | -6.2 (-8.7, -3.7 ) | -6.1 (-8.6,-3.5 ) |
| **Unemployed** |  | -3.2 (-5.0, -1.3 ) | -3.1 (-4.9, -1.2 ) |
| **Age (per decade)** |  | -0.73 (-1.1,-0.38) | -0.77 (-1.1, -0.42) |
| **Sex (Male^b^)** |  |  |  |
| **Female** |  | -1.9 (-2.7,-1.0 ) | -1.8 (-2.6, -0.91) |
| **Injury cause (Road traffic^b^)** |  |  |  |
| **Incidental fall** |  |  | 0.91 (-0.00, 1.8 ) |
| **Other non-intentional injury** |  |  | -0.58 (-0.90, 2.1 ) |
| **Violence or Assault** |  |  | -0.20(-2.3, 1.9 ) |
| **Suicide attempt** |  |  | -1.0 (-5.3, 3.2 ) |
| **Pre-injury substance abuse (No^b^)** |  |  |  |
| **Yes** |  |  | 2.3 (-0.36, 4.9 ) |
| **Pre-injury mental health problems (No^b^)** |  |  |  |
| **Yes** |  |  | -1.1 (-2.5, 0.27) |
| **Living arrangement (Together^b^)** |  |  |  |
| **Alone** |  |  | -0.71 (-1.7, 0.27) |
| **R^2^ development cohort** | 0.14 | 0.21 | 0.22 |
| **Optimism** | 0.01^c^ | 0.01 | 0.02 |
| **R^2^ after bootstrap validation** | - | 0.20 | 0.20 |

Note: ^a^ The models were fitted with additional imputed six months outcome whenever three or twelve months outcomes were available.

^b^ Reference category of categorical variable.

^c^ Optimism of the core model is estimated to be similar to that of the extended model.

Core model = Glasgow Coma Scale, Major extracranial injury and pre-injury health status (ASA-PS).

Extended model = Core plus education, employment and sex.

Full model = Extended plus injury cause, history of substance abuse, history of mental health problems, and living arrangement.
