# Supplementary material for: Development of prognostic models for Health-Related Quality of Life following traumatic brain injury
Source: Qual Life Res. 2021 Jul 30;31(2):451–71. doi: 10.1007/s11136-021-02932-z (PMC8847302; doi:10.1007/s11136-021-02932-z)
Supplement: Supplementary file 10 — Supplementary file10 (DOCX 16 kb) [file 11136_2021_2932_MOESM10_ESM.docx]

**Supplementary Table 3** *Regression coefficients and 95% confidence intervals for the SF-36v2 mental health component summary score (MCS) with multivariable linear regression analysis. Model performance indicated by explained variance (R^2^) and bootstrap validation for each model (N=2535^a^).*

| *MCS* | **Core Model** | **Extended Model** | **Full Model** |
| --- | --- | --- | --- |
| **Predictors** |  |  |  |
| **Constant** | 49 | 45 | 44 |
| **Pre-injury mental health problems (No^b^)** |  |  |  |
| **Yes** | -7.9 (-9.6,-6.3 ) | -7.1 (-8.8, -5.5 ) | -7.1 (-8.8,-5.5 ) |
| **Education (College/Uni degree^b^)** |  |  |  |
| **Currently in school** | -1.7 (-3.1,-0.30) | -1.7 (-3.1,-0.32) | -1.7 (-3.1,-0.31 ) |
| **None/Primary school** | -4.5 (-6.2,-2.8 ) | -4.4 (-6.1,-2.8 ) | -4.4 (-6.1,-2.8 ) |
| **Secondary/high school** | -1.0(-2.2, 0.25) | -0.85(-2.1, 0.40) | -0.84(-2.1, 0.41) |
| **Employment (Working^b^)** |  |  |  |
| **Homemaker** | -4.9 (-9.0,-0.71) | -3.6 (-7.5, 0.86) | -3.7 (-8.0, 0.52) |
| **Student** | -0.35(-2.2, 1.5 ) | -0.41(-2.1, 1.7 ) | -0.21(-2.3, 1.9) |
| **Retired** | 2.1 (0.95, 3.3 ) | 2.4 ( 1.1, 3.7 ) | 2.2 ( 0.62, 3.8) |
| **Unable to work/sick leave** | -6.0 (-8.9,-3.0) | -4.9 (-7.9,-1.9 ) | -5.0 (-8.0,-2.0 ) |
| **Unemployed** | -3.1 (-5.5,-0.83) | -3.0 (-5.3,-0.70) | -3.0 (-5.3,-0.67) |
| **Injury cause (Road traffic^b^)** |  |  |  |
| **Incidental fall** |  | 2.2 ( 1.1, 3.3 ) | 2.2 ( 1.1, 3.2 ) |
| **Other non-intentional injury** |  | 1.5 (-0.32, 3.2) | 1.4 (-0.33, 3.2) |
| **Violence or Assault** |  | 0.12(-2.3, 2.5 ) | 0.17 (-2.2, 2.6 ) |
| **Suicide attempt** |  | 5.0 (0.01, 9.9) | 5.0 (-0.2, 9.9 ) |
| **GCS** |  | 0.24(0.12, 0.35) | 0.23(0.12,0.35) |
| **ASA-PS (Healthy patient^b^)** |  |  |  |
| **Mild systemic disease** |  | -0.91(-2.0, 0.19) | -0.97 (-2.1,0.17) |
| **Severe systemic disease** |  | -3.2 (-5.0, -1.3 ) | -3.2 (-5.1,-1.3 ) |
| **Pre-injury substance abuse (No^b^)** |  |  |  |
| **Yes** |  | -4.1 (-7.3,-0.84) | -4.0 (-7.3,-0.77) |
| **Sex (Male^a^)** |  |  |  |
| **Female** |  | -1.6 (-2.7,-0.62) | -1.7 (-2.7,-0.63) |
| **Living arrangement (Together^a^)** |  |  |  |
| **Alone** |  | -1.3 (-2.5,-0.13) | -1.3 (-2.5,-0.14) |
| **Mei (No^b^)** |  |  |  |
| **Yes** |  | -0.92(-2.0, 0.13) | -0.92(-2.0, 0.14) |
| **Age (per decade)** |  |  | 0.09(-0.33,0.52) |
| **R^2^ development cohort** | 0.08 | 0.10 | 0.11 |
| **R^2^ optimism** | 0.01^c^ | 0.01 | 0.02 |
| **R^2^ after bootstrap validation** | - | 0.09 | 0.09 |

Note: ^a^ The models were fitted with additional imputed six months outcome whenever three or twelve months outcomes were available.

^b^ Reference category of categorical variable.

^c^ Optimism of the core model is estimated to be similar to that of the extended model

Core model = History of mental health problems, education and employment.

Extended model = Core plus injury cause, GCS, ASA-PS, living arrangement, MEI and sex.

Full model = Extended plus age.
