# Supplementary material for: Development of prognostic models for Health-Related Quality of Life following traumatic brain injury
Source: Qual Life Res. 2021 Jul 30;31(2):451–71. doi: 10.1007/s11136-021-02932-z (PMC8847302; doi:10.1007/s11136-021-02932-z)
Supplement: Supplementary file 11 — Supplementary file11 (DOCX 16 kb) [file 11136_2021_2932_MOESM11_ESM.docx]

| *QoLIBRI* | **Core Model** | **Extended Model** | **Full Model** |
| --- | --- | --- | --- |
| **Predictors** |  |  |  |
| **Constant** | 78 | 72 | 72 |
| **Pre-injury mental health problems (No^b^)** |  |  |  |
| **Yes** | -9.9 (-12.0,-7.4 ) | -9.1 (-12.0, -6.6) | -8.8 (-11.0,-6.2 ) |
| **Education (College/Uni degree^b^)** |  |  |  |
| **Currently in school** | -4.8 (-6.9, -2.7) | -4.6 (-6.7,-2.4) | -4.6 (-6.7, -2.4 ) |
| **None/Primary school** | -11.0(-14.0,-8.8) | -11.0(-13.0,-8.2) | -11.0(-13.0,-8.2) |
| **Secondary/high school** | -5.0 (-6.9, -3.0) | -4.6 (-6.5, -2.7 ) | -4.5 (-6.5, -2.6 ) |
| **Employment (Working^b^)** |  |  |  |
| **Homemaker** | -11.0(-18, -4.7) | -8.8 (-15.0,-2.3) | -8.6(-15.0,-2.2 ) |
| **Student** | 1.6 (-1.3, 4.5 ) | -0.08(-3.3, 3.1 ) | -0.13 (-3.3, 3.0 ) |
| **Retired** | -0.40 (-2.2, 1.4 ) | 1.9 (-0.57,4.4 ) | 2.1 (-0.41,4.5) |
| **Unable to work/sick leave** | -11.0(-16.0,-6.6) | -9.5 (-14.0,-4.9) | -9.0 (-14.0,-4.4 ) |
| **Unemployed** | -8.2 (-12.0,-4.7) | -8.3 (-12.0,-4.9) | -8.1 (-12.0, -4.6) |
| **Injury cause (Road traffic^b^)** |  |  |  |
| **Incidental fall** |  | 3.1 ( 1.4, 4.8 ) | 3.1 ( 1.5, 4.8 ) |
| **Other non-intentional injury** |  | 3.2 ( 0.46, 5.9) | 3.2 ( 0.46, 5.9 ) |
| **Violence or Assault** |  | -1.2 (-4.9, 2.5 ) | -1.1 (-4.8, 2.6 ) |
| **Suicide attempt** |  | 3.4 (-4.2, 11.0) | 3.5 (-4.1, 11.0) |
| **GCS** |  | 0.63(0.45, 0.81) | 0.62 (0.44,0.80) |
| **ASA-PS (Healthy patient^b^)** |  |  |  |
| **Mild systemic disease** |  | -2.1 (-3.8,-0.31) | -1.9 (-3.7,-0.18 ) |
| **Severe systemic disease** |  | -7.5 (-10.0,-4.6) | -7.2 (-10.0,-4.3 ) |
| **Pre-injury substance abuse (No^b^)** |  |  |  |
| **Yes** |  |  | -3.5 (-8.4, 1.5 ) |
| **Sex (Male^b^)** |  |  |  |
| **Female** |  | -1.9 (-3.5,-0.36) | -1.9(-3.5,-0.33 ) |
| **Living arrangement (Together^b^)** |  |  |  |
| **Alone** |  |  | -1.4 (-3.2, 0.42) |
| **Mei (No^b^)** |  |  |  |
| **Yes** |  | -2.8 (-4.4,-1.2 ) | -2.9 (-4.5, -1.3 ) |
| **Age (per decade)** |  | -0.69(-1.3,-0.04) | -0.71(-1.4,-0.06) |
| **R^2^ development cohort** | 0.10 | 0.15 | 0.15 |
| **R^2^ optimism** | 0.02^c^ | 0.02 | 0.02 |
| **R^2^ after bootstrap validation** | - | 0.13 | 0.13 |

**Supplementary Table 4** *Regression coefficients and 95% confidence intervals for the Quality of Life after Traumatic Brain Injury (QoLIBRI) total score with multivariable linear regression analysis. Model performance indicated by explained variance (R^2^) and bootstrap validation for each model (N=2535^a^)*

Note: ^a^ The models were fitted with additional imputed six months outcome whenever three or twelve months outcomes were available.

^b^ Reference category of categorical variable.

^c^ Optimism of the core model is estimated to be similar to that of the extended model.

Core model = Education, employment type and pre-injury mental health problems.

Extended model = Core plus injury cause, GCS, ASA-PS, sex, MEI, and age.

Full model = Extended plus pre-injury substance abuse, and living arrangement.
