# Supplementary material for: Development of prognostic models for Health-Related Quality of Life following traumatic brain injury
Source: Qual Life Res. 2021 Jul 30;31(2):451–71. doi: 10.1007/s11136-021-02932-z (PMC8847302; doi:10.1007/s11136-021-02932-z)
Supplement: Supplementary file 12 — Supplementary file12 (DOCX 14 kb) [file 11136_2021_2932_MOESM12_ESM.docx]

| *PCS* | **Full model** | **χ2–2df^b^** |
| --- | --- | --- |
| **Constant** | 0.25 |  |
| **Predictors** |  |  |
| **GCS** | 0.84 (0.79, 0.90) | 27 |
| **MEI (No^a^)** |  | 25 |
| **Yes** | 2.0 (1.5, 2.6) |  |
| **ASA-PS (Healthy patient^a^)** |  | 25 |
| **Mild systemic disease** | 1.5 (1.1, 2.0) |  |
| **Severe systemic disease** | 3.2 (2.1, 4.8) |  |
| **Education (College/Uni degree^a^)** |  | 10 |
| **Currently in school** | 1.4 (0.96, 1.90) |  |
| **None/Primary school** | 2.2 (1.5, 3.2) |  |
| **Secondary/high school** | 1.4 (1.0, 2.0) |  |
| **Employment (Working^a^)** |  | 18 |
| **Homemaker** | 3.7 (1.5, 9.4) |  |
| **Student** | 0.90 (0.50, 1.6) |  |
| **Retired** | 1.3 (0.90, 1.9) |  |
| **Unable to work/sick leave** | 3.1 (1.6, 5.8) |  |
| **Unemployed** | 2.5 (1.5, 4.1) |  |
| **Age (per decade)** | 1.45 (1.0, 2.1) | 2 |
| **Sex (Male^a^)** |  | 9 |
| **Female** | 1.53 (1.2, 2.0) |  |
| **Injury cause (Road traffic^a^)** |  | -5 |
| **Incidental fall** | 1.2 (0.88, 1.5) |  |
| **Other non-intentional injury** | 1.4 (0.87, 2.2) |  |
| **Violence/Assault** | 1.2 (0.59, 2.3) |  |
| **Suicide attempt** | 1.5 (0.52, 4.4) |  |
| **Pre-injury substance abuse (No^a^)** |  | -1 |
| **Yes** | 0.61 (0.26, 1.4) |  |
| **Pre-injury mental health problems (No^a^)** |  | 5 |
| **Yes** | 1.7 (1.2, 2.5) |  |
| **Living arrangement (Together^a^)** |  | -1 |
| **Alone** | 1.2 (0.87, 1.5) |  |

**Supplementary Table 5** *Regression coefficients and 95% confidence intervals for impaired SF-36v2 physical health component summary score (<40) with multivariable logistic regression analysis (N=1726; 437 with PCS < 40).*

*Note: ^a^ Reference category of categorical variable.*

*^b^ The strength of predictors was based on the likelihood ratio χ2 test statistic minus twice the degrees of freedom, which gives a fair assessment of a factor’s predictiveness.*
