# Supplementary material for: Development of prognostic models for Health-Related Quality of Life following traumatic brain injury
Source: Qual Life Res. 2021 Jul 30;31(2):451–71. doi: 10.1007/s11136-021-02932-z (PMC8847302; doi:10.1007/s11136-021-02932-z)
Supplement: Supplementary file 13 — Supplementary file13 (DOCX 14 kb) [file 11136_2021_2932_MOESM13_ESM.docx]

**Supplementary Table 6** *Regression coefficients and 95% confidence intervals for impaired SF-36v2 mental health component summary score (<40) with multivariable logistic regression analysis (N=1727, 472 with MCS <40).*

| *MCS* | **Full model** | **χ^2^–2df ^b^** |
| --- | --- | --- |
| **Constant** | 0.48 |  |
| **Predictors** |  |  |
| **GCS** | 0.91 (0.85, 0.97) | 7 |
| **MEI (No^a^)** |  |  |
| **Yes** | 1.04 (0.80, 1.3) | -2 |
| **ASA-PS (Healthy patient^a^)** |  | 4 |
| **Mild systemic disease** | 1.2 (0.91, 1.6) |  |
| **Severe systemic disease** | 1.9 (1.2, 2.9) |  |
| **Education (College/Uni degree^a^)** |  | 12 |
| **Currently in school** | 1.5 (1.1, 2.1) |  |
| **None/Primary school** | 2.2 (1.5, 3.2) |  |
| **Secondary/high school** | 1.3 (0.9, 1.7) |  |
| **Employment (Working^a^)** |  | 25 |
| **Homemaker** | 3.1 (1.2, 7.6) |  |
| **Student** | 0.98 (0.60, 1.6) |  |
| **Retired** | 0.68 (0.46, 1.0) |  |
| **Unable to work/sick leave** | 2.99 (1.6, 5.8) |  |
| **Unemployed** | 2.2 (1.3, 3.5) |  |
| **Age (per decade)** | 1.1 (0.78, 1.5) | -2 |
| **Sex (Male^a^)** |  | 4 |
| **Female** | 1.4 (1.1, 1.7) |  |
| **Injury cause (Road traffic^a^)** |  | 6 |
| **Incidental fall** | 1.5 (1.2, 1.9) |  |
| **Other non-intentional injury** | 0.99 (0.62, 1.6) |  |
| **Violence/Assault** | 1.7 (0.94, 3.0) |  |
| **Suicide attempt** | 0.47 (0.15, 1.5) |  |
| **Pre-injury substance abuse (No^a^)** |  | 5 |
| **Yes** | 2.9 (1.3, 6.3) |  |
| **Pre-injury mental health problems (No^a^)** |  | 32 |
| **Yes** | 2.9 (2.0, 4.0) |  |
| **Living arrangement (Together^a^)** |  | 2 |
| **Alone** | 1.3 (1.0, 1.8) |  |

Note: ^a^ Reference category of categorical variable.

^b^ The strength of predictors was based on the likelihood ratio χ2 test statistic minus twice the degrees of freedom, which gives a fair assessment of a factor’s predictiveness.
