# Supplementary material for: Development of prognostic models for Health-Related Quality of Life following traumatic brain injury
Source: Qual Life Res. 2021 Jul 30;31(2):451–71. doi: 10.1007/s11136-021-02932-z (PMC8847302; doi:10.1007/s11136-021-02932-z)
Supplement: Supplementary file 14 — Supplementary file14 (DOCX 14 kb) [file 11136_2021_2932_MOESM14_ESM.docx]

| *QoLIBRI* | **Full model** | **χ2–2df^b^** |
| --- | --- | --- |
| **Constant** | 0.36 |  |
| **Predictors** |  |  |
| **GCS** | 0.84 (0.79, 0.89) | 29 |
| **MEI (No^a^)** |  | 1 |
| **Yes** | 1.3 (0.97, 1.6) |  |
| **ASA-PS (Healthy patient^a^)** |  | 8 |
| **Mild systemic disease** | 1.3 (0.97, 1.7) |  |
| **Severe systemic disease** | 2.2 (1.4, 3.4) |  |
| **Education (College/Uni degree^a^)** |  | 24 |
| **Currently in school** | 1.7 (1.2, 2.4) |  |
| **None/Primary school** | 3.0 (2.0, 4.4) |  |
| **Secondary/high school** | 1.5 (1.1, 2.1) |  |
| **Employment (Working^a^)** |  | 23 |
| **Homemaker** | 2.3 (0.93, 5.6) |  |
| **Student** | 0.92 (0.53, 1.6) |  |
| **Retired** | 0.67 (0.45, 0.99) |  |
| **Unable to work/sick leave** | 2.0 (1.0, 3.7) |  |
| **Unemployed** | 2.8 (1.7, 4.7) |  |
| **Age (per decade)** | 1.4 (1.0, 2.0) | 2 |
| **Sex (Male^a^)** |  | 8 |
| **Female** | 1.5 (1.2, 2.0) |  |
| **Injury cause (Road traffic^a^)** |  | 2 |
| **Incidental fall** | 0.73 (0.56, 0.96) |  |
| **Other non-intentional injury** | 0.78 (0.48, 1.3) |  |
| **Violence/Assault** | 1.7 (0.94, 3.1) |  |
| **Suicide attempt** | 0.81 (0.28, 2.4) |  |
| **Pre-injury substance abuse (No^a^)** |  | -1 |
| **Yes** | 1.6 (0.72, 3.7) |  |
| **Pre-injury mental health problems (No^a^)** |  | 11 |
| **Yes** | 2.0 (1.4, 2.8) |  |
| **Living arrangement (Together^a^)** |  | -1 |
| **Alone** | 1.2 (0.88, 1.6) |  |

**Supplementary Table 7** *Regression coefficients and 95% confidence intervals for impaired Quality of Life after Traumatic Brain Injury* *total score (<60) with multivariable logistic regression analysis (N= 1723, 410 with QoLIBRI total score < 60).*

Note: ^a^ Reference category of categorical variable.

^B^ The strength of predictors was based on the likelihood ratio χ2 test statistic minus twice the degrees of freedom, which gives a fair assessment of a factor’s predictiveness.
