# Supplementary material for: Development of prognostic models for Health-Related Quality of Life following traumatic brain injury
Source: Qual Life Res. 2021 Jul 30;31(2):451–71. doi: 10.1007/s11136-021-02932-z (PMC8847302; doi:10.1007/s11136-021-02932-z)
Supplement: Supplementary file 15 — Supplementary file15 (DOCX 14 kb) [file 11136_2021_2932_MOESM15_ESM.docx]

| **Supplementary Table 8** Formulas of Health-Related Quality of Life models for the SF-36v2 physical (PCS) and mental (MCS) component summary score and the Quality of Life after Traumatic Brain Injury (QoLIBRI) total score six months post-injury | |
| --- | --- |
| *PCS* | |
| **Core model** | $46.0 +0.35\times GCS +-3.7 \times MEI + -4.0 \times mild systemic disease + -10 \times severe systemic disease$ |
| **Extended model** | $49.0 +0.38\times GCS +-4.2 \times MEI + -2.0 \times mild systemic disease + -7.2 \times severe systemic disease +-1.7 \times currently in school +-4.3 \times none or primary school + -1.5 secondary or high school +-4.4 \times homemaker +0.41\times student +-1.3 \times retired +-6.3\times unable to work or sick leave + -3.2\times unemployed +-0.73 \times age per decade + -2.1 \times female sex$ |
| **Full model** | $49.0 +0.39\times GCS+ -4.1 \times MEI + -2.0 \times mild systemic disease + -7.3 \times severe systemic disease+ -1.8 \times currently in school+ -4.3 \times none or primary school + -1.6 secondary or high school +-4.6 \times homemaker +0.45\times student +-1.4 \times retired +-6.1\times unable to work or sick leave + -3.0\times unemployed +-0.74 \times age per decade + -2.0 \times female sex +-0.71\times Incidental fall +-0.50\times other non intentional injury +-0.18\times violence or assualt +-1.4\times suicide attempt +3.2\times pre-injury substance abuse +-1.2\times pre-injury mental health problems +-0.87\times living alone$ |
| *MCS* | |
| **Core model** | $49.0 +-7.5 pre-injury mental health problems +-1.7\times currently in school +-4.4\times none or primary school +-0.96\times secondary or high school +-6.4\times homemaker +-0.33\times student +2.1\times retired +-5.8 +-4.1\times unemployed$ |
| **Extended model** | $45.0+-6.9\times pre-injury mental health problem+-1.8\times currently in school+-4.3\times none or primary school+-0.85\times secondary or high school+-4.4\times homemaker+-0.48\times student +2.5\times retired+-4.5 \times unable to work or sick leave +-4.0\times unemployed +2.2 \times incidental fall+1.2 \times non intentional injury +0.01 \times violence or assualt +4.9 \times suicide attempt +0.22 \times GCS+-0.95\times mild systemic disease+ -3.4 \times severe systemic disease +-4.4 \times pre-injury substance abuse +-2.1 \times female sex+-1.3 \times living alone+ -1.2 \times MEI$ |
| **Full model** | $40.0+ -6.8\times pre-injurymental health problems+ -1.8\times currently in school +-4.4\times none or primary school +-0.84\times secondary or high school +-4.5\times homemaker +-0.31\times student +2.3\times retired +-4.6 \times unable to work or sick leave+-4.0\times unemployed +2.2 \times incidental fall+1.2 \times non intentional injury+ -0.04 \times violence or assualt +4.9 \times suicide attempt +0.22 \times GCS+-1.0\times mild systemic disease+ -3.5 severe systemic disease +-4.3 pre-injury substance abuse +-2.1 female sex +-1.3 living alone +-1.2 MEI +0.08 age per decade$ |
| *QoLIBRI* | |
| **Core model** | $78.0 +9.8 pre-injury mental health problems +-5.1\times currently in school +-11.0\times none or primary school +-4.8\times secondary or high school +-12.0\times homemaker +-1.6\times student +-0.30\times retired +-11.0 \times unable to work or sick leave+-9.4\times unemployed$ |
| **Extended model** | $70.0 +-9.0\times pre-injury mental health problems+-5.0\times currently in school +-10.0\times none or primary school +-4.4\times secondary or high school +-10.0\times homemaker +-1.3\times student +0.47\times retired +-9.4 \times unable to work or sick leave+-9.1\times unemployed +2.8 \times incidental fall+3.2 \times non intentional injury+ -1.0 violence or assualt +3.1 \times suicide attempt +0.56 \times GCS+-2.4\times mild systemic disease+ -8.9 severe systemic disease +-2.4 \times female sex+ -3.1 \times MEI+ -0.62 \times age per decade$ |
| **Full model** | $73.0+-8.8\times pre-injury mental health problems+ -5.0\times currently in school +-10.0\times none or primary school +-4.5\times secondary or high school +-9.1\times homemaker +-0.11\times student +2.0\times retired +-8.6 \times unable to work or sick leave+-9.2\times unemployed +3.1 \times incidental fall+3.3 \times non intentional injury+ - 1.2 violence or assualt +3.2 \times suicide attempt +0.57 \times GCS+-1.9\times mild systemic disease+ -8.1 severe systemic disease +-2.9 pre-injury substance abuse +-2.3 female sex +-1.2 living alone +-3.2 MEI +0.63 \times age per decade$ |
